# Using the Hierarchies of Evidence Applied to Lifestyle Medicine (HEALM) Approach to Assess the Strength of Evidence on Associations between Dietary Patterns and All-Cause Mortality

**DOI:** 10.3390/nu14204340

**Published:** 2022-10-17

**Authors:** Kate Wingrove, Mark A. Lawrence, Priscila Machado, Lena D. Stephens, Sarah A. McNaughton

**Affiliations:** Institute for Physical Activity and Nutrition (IPAN), School of Exercise and Nutrition Sciences, Deakin University, Geelong 3220, Australia

**Keywords:** dietary patterns, dietary guidelines, evidence synthesis, evidence translation, HEALM, GRADE

## Abstract

Dietary guidelines should be underpinned by high-quality evidence. Quality assessment methods that reflect traditional evidence hierarchies prioritise evidence from randomised controlled trials (RCTs). The Hierarchies of Evidence Applied to Lifestyle Medicine (HEALM) approach is an alternative quality assessment method for research questions that for practical and/or ethical reasons, cannot be answered using RCTs. The aim of this study was to investigate how the HEALM approach could be used to assess the strength of evidence on associations between dietary patterns and all-cause mortality (a research question that is difficult to answer using RCTs). Two data sources were used: an existing systematic review of dietary patterns and all-cause mortality that synthesised evidence from observational studies; and an overview of reviews that was conducted to summarise relevant evidence from mechanistic and intervention studies. A set of four criteria were developed and used in the application of HEALM. Using different datasets in combination, the strength of evidence was rated as ‘Grade B: moderate/suggestive’ or ‘Grade C: insufficient/inconclusive’. HEALM is a novel approach for integrating and assessing the strength of evidence from mechanistic, intervention, and observational studies. Further research is needed to address the practical challenges that were identified in the application of HEALM.

## 1. Introduction

Since the 1980s, evidence on associations between dietary patterns and a range of health outcomes has been mounting [1,2,3]. As exposures, dietary patterns encompass the complex interactions between the combinations and amounts of foods that are frequently consumed, and the nutrients (and other components) those foods contain [2,4,5]. Dietary patterns evidence is derived primarily from prospective cohort studies where participants are followed over extended periods of time [6,7,8]. These observational studies allow associations with long-term health outcomes (e.g., chronic disease incidence, all-cause mortality) to be assessed [9,10,11]. Randomised controlled trials (RCTs) of dietary pattern interventions do exist, but these studies tend on focus on intermediate outcomes where impacts can be observed in shorter periods of time (e.g., blood pressure, blood lipids, body weight) [12,13].

It is now well accepted that dietary guidelines should be underpinned by systematic reviews [14,15,16], including systematic reviews of dietary patterns evidence [12,17,18]. Cochrane provides guidelines for conducting systematic reviews, and the World Health Organization (WHO) provides guidelines for the use of systematic reviews in guideline development [19,20]. The first step in the systematic review process is to define the research question in terms of the Population, Intervention (or exposure), Comparator, and Outcome of interest (PICO). Once relevant studies have been identified and data have been extracted from included studies, the risk of bias (ROB) associated with each study is assessed, the data from included studies are synthesised (using a meta-analysis where possible), the quality (or certainty) of the evidence for each outcome is assessed, and conclusions are drawn [19,20]. In guideline development, the quality of the evidence for each outcome is assessed alongside other criteria (e.g., risk of unintended benefits and harms) to inform recommendations [21,22].

To assess the quality (or certainty) of the evidence, Cochrane and WHO recommend using the Grading of Recommendations, Assessment, Development and Evaluation (GRADE) approach [19,20,23]. Using GRADE, the certainty of evidence from RCTs is initially considered ‘high’, and can be downgraded based on risk of bias, inconsistency, indirectness, imprecision, or publication bias [24,25]. In contrast, the certainty of evidence from non-randomised studies (including cohort studies) is initially considered ‘low’. It can be downgraded applying the same criteria. There is also potential for evidence from cohort studies to be upgraded based on additional criteria, including a large effect size or an observed dose–response gradient [24,25]. Using GRADE, the overall ‘certainty’ of the evidence is rated as ‘high’, ‘moderate’, ‘low’, or ‘very low’.

GRADE is a systematic and transparent approach, and its integration with Cochrane tools for assessing the risk of bias associated with individual studies (ROB2 for randomised studies and ROBINS-I for non-randomised studies) enables systematic review authors to assess and report the quality of the evidence in a consistent way [26,27]. However, GRADE reflects a traditional evidence hierarchy, where evidence from randomised studies is rated more highly than evidence from non-randomised studies from the outset, regardless of the research question being addressed [24,25]. For research questions that cannot be addressed using RCTs for practical and/or ethical reasons, including questions on associations between dietary patterns and long-term health outcomes, the best available evidence is likely to be derived from large prospective cohort studies that are conducted over years or decades [7,13]. Using GRADE, the certainty of this evidence is unlikely to be ‘high’, and therefore, may not be considered for translation into dietary guidelines (despite being highly relevant).

To address these concerns, alternative approaches have been developed for research questions that are difficult to address using evidence from RCTs. For example, the World Cancer Research Fund (WCRF) developed their own approach for ‘judging the evidence’ on associations between diet and cancer-related outcomes (including incidence and mortality) [7]. The WCRF approach reflects the Bradford Hill criteria, which suggest that data from observational studies can be used to infer causality (provided evidence to support biological plausibility also exists) [7]. More recently, the Hierarchies of Evidence Applied to Lifestyle Medicine (HEALM) approach was proposed by Katz et al. [28]. The authors of HEALM present an ‘evidence threshold pathway mapping’ process to determine the most suitable quality assessment method based on whether the research question is ‘definitively addressable with RCTs’ and if so, whether RCTs have been conducted [28]. If the answer to both of these questions is yes, the recommendation is to use GRADE. A research question is not ‘definitively addressable with RCTs’ if “a duration of > 5 years adherence to the intervention is required, randomization is not plausible or ethical, exposure of interest is the cumulative, lifetime effect of health behaviours” in which case, the recommendation is to consider HEALM [28]. The application of HEALM requires evidence from mechanistic studies, intervention studies and observational studies to be assessed. The overall ‘strength’ of the evidence is rated as ‘Grade A: strong/decisive’, ‘Grade B: moderate/suggestive’, or ‘Grade C: insufficient/inconclusive’.

HEALM is a relatively new approach with limited application in the field to date. Only one systematic review that used HEALM has been published [29]. In that review, the authors examined a total of 28 cohort studies on associations between fruit and vegetable intake, cancer recurrence and mortality, and all-cause mortality in cancer patients [29]. However, a lack of detail was provided on the application of the method to allow replication by other researchers and guideline developers. HEALM is recommended for research questions that cannot be answered using RCTs, yet the application of HEALM requires evidence from mechanistic and intervention studies to be assessed [28]. This raises some important methodological questions regarding the application of HEALM. For example, it is unclear what constitutes relevant evidence from mechanistic and intervention studies, and how relevant evidence should be identified and analysed. Therefore, the aim of this study was to investigate how the HEALM approach could be used to assess the strength of evidence on associations between dietary patterns and all-cause mortality. The focus on dietary patterns and all-cause mortality was selected as an example of a research question that is difficult to address using RCTs, and one that has been asked the context of dietary guideline development [4,30].

## 2. Materials and Methods

### 2.1. Overview

An overview of the HEALM approach (as described by the creators of HEALM) is provided in Table 1 [28]. Four questions (Q1-4) need to be answered using evidence from mechanistic studies, intervention studies, and observational studies. The responses to Q1-4 are then used to calculate an overall score and rate the overall strength of evidence [28].

For this study, two data sources were used: an existing systematic review of dietary patterns and all-cause mortality that synthesised evidence from observational studies; and an overview of reviews that was conducted specifically for this study to summarise relevant evidence from mechanistic and intervention studies. A description of these data sources and the criteria that were used in the application of HEALM is provided in the sections that follow.

### 2.2. Systematic Review

The objective of the systematic review was to synthesise evidence on associations between dietary patterns and all-cause mortality in adults. The systematic review was commissioned by the WHO to inform dietary guidelines and was conducted by the authors of this study [3,30].

#### 2.2.1. Eligibility Criteria

An overview of the eligibility criteria is provided in Table S1. RCTs, prospective cohort studies, and nested case–control studies published in any language between January 1980 and March 2019 were eligible for inclusion. The population of interest was healthy, free-living adults (aged over 18 years). Dietary patterns assessed using index-based methods, factor analysis or principal component analysis (FA/PCA), cluster analysis (CA), or reduced rank regression (RRR) were the interventions/comparators of interest. For RCTs, interventions that included two or more food groups consumed together as part of a dietary pattern were also eligible for inclusion [30].

#### 2.2.2. Search Strategy and Selection Process

Three databases were searched in March 2019 (PubMed, EMBASE, and Global Health) The search strategy combined a set of terms related to study design (e.g., RCT, cohort, nested case–control) with a set of terms related to dietary patterns and dietary patterns assessment methods (e.g., dietary pattern, Mediterranean diet, factor analysis). Filters were used to limit the search to studies conducted in humans and reported in academic journal articles published between January 1980 and March 2019. No language limits were applied. Screening was conducted independently by two authors and discrepancies were resolved via discussion [30].

#### 2.2.3. Data Collection Process and Data Items

Data extraction was conducted independently by two authors and discrepancies were resolved by a third author. Data items included study characteristics (e.g., study design, inclusion criteria, sample size, participant characteristics), dietary intake assessment methods (e.g food frequency questionnaire, 24 h recall), dietary pattern assessment methods (e.g., Healthy Eating Index, reduced rank regression), and relative measures of effect size for each outcome measure (e.g., Relative Risk, Hazard Ratio, Risk Ratio) [30].

#### 2.2.4. Risk of Bias Assessment

ROB associated with individual studies was assessed using ROBINS-I. ROB assessments were conducted independently by two authors and discrepancies were resolved via discussion [30].

#### 2.2.5. Synthesis Methods

Data were synthesised using meta-analyses where possible. Data from studies that met the inclusion criteria but were unable to be included in a meta-analysis were included in a narrative synthesis [30].

#### 2.2.6. Certainty Assessment

The certainty of the evidence included in each meta-analysis and each narrative synthesis was assessed using GRADE [30]. GRADE was applied independently by two authors and disagreements were discussed until consensus was reached.

#### 2.2.7. Use of Systematic Review Data

A total of 78 prospective cohort studies were included in the systematic review [30]. While RCTs were eligible for inclusion, none were identified (as expected based on the research question). To account for variation between studies in terms of the dietary pattern assessment method that was used (index-based methods, FA/PCA, CA, or RRR) and the type of outcome data that was provided (continuous or categorical), a total of five meta-analyses and four narrative syntheses were conducted. For the purpose of this study, data that had previously been extracted from the studies included in each meta-analysis and each narrative synthesis were collated and summarised using descriptive statistics (Table S2).

### 2.3. Overview of Reviews

The objective of the overview of reviews was to summarise relevant evidence from systematic and narrative reviews on (1) mechanisms of action and (2) effects of interventions relevant to dietary patterns and all-cause mortality. Methods consistent with Cochrane were applied where relevant [31].

#### 2.3.1. Eligibility Criteria

The eligibility criteria for the overview of reviews were adapted from the criteria for the systematic review (Table S1). The interventions/comparators of interest were expanded to include foods, nutrients, other food components, and supplements, because mechanisms of action relate to nutrients and other food components that are consumed as part of dietary patterns, and because evidence from supplement trials can provide evidence of plausible biological mechanisms [7,17]. The outcome of interest was expanded to include any health outcome, because there are many possible interim measures for all-cause mortality, including chronic disease risk factors and chronic disease incidence [32,33].

The population of interest was expanded to include animal models and cell models (for mechanistic studies) and humans including adults and children with and without disease (for intervention studies) to capture evidence on a broader range of interventions/comparators and outcomes. The 1980 start date was removed because this start date was specific to the emergence of dietary patterns evidence [2,34].

#### 2.3.2. Search Strategy and Selection Process

To identify reviews that included at least three relevant mechanistic and/or intervention studies, reference lists of a random sample of prospective cohort studies included in the systematic review were hand searched by one author (KW). Of the 78 included studies, 64 studies used index-based dietary pattern assessment methods and 16 studies used data driven methods (FA/PCA, CA, or RRR) (Figure S1). Some studies used a combination of index-based and data driven methods. To account for possible differences in the reviews that were cited, a 20% sample was identified from a list of studies that used index-based methods (n = 12) using the random number generation function in Excel. This process was repeated to identify a 20% sample of studies that used data driven methods (n = 3).

#### 2.3.3. Data Collection Process and Data Items

Data extraction was conducted independently by one author (KW or LDS). Data items included review type and the number and characteristics of relevant mechanistic and interventions studies included in each review (Table S2).

#### 2.3.4. Synthesis Methods

Descriptive Statistics were used to summarise the data (Table S2).

### 2.4. Criteria Developed and Used in the Application of HEALM

#### 2.4.1. Development of Criteria

An overview of the criteria that were developed and used in the application of HEALM is provided in Table 2. For each of the four questions (Q1-4), four criteria (quantity, quality, consistency, and significance) were developed based on the information provided by the authors of HEALM as outlined in Table 1 [28] in combination with information from other sources where needed [23,35].

#### 2.4.2. Strength of Evidence Assessment

The strength of the evidence was assessed independently by two authors (KW and PM) using different combinations of data from each data source (the systematic review and the overview of reviews). Disagreements were discussed until consensus was reached, and comments were added to explain the decisions that were made.

#### 2.4.3. Comparison to GRADE

The overall strength of evidence (assessed using HEALM) was compared to the certainty of evidence (assessed using GRADE). HEALM was used to assess evidence from mechanistic, intervention, and observational studies (using data from the overview of reviews and the systematic review), whereas GRADE was used by systematic review authors to assess evidence from observational studies included in each meta-analysis and narrative synthesis [30].

## 3. Results

**Figure 1:**
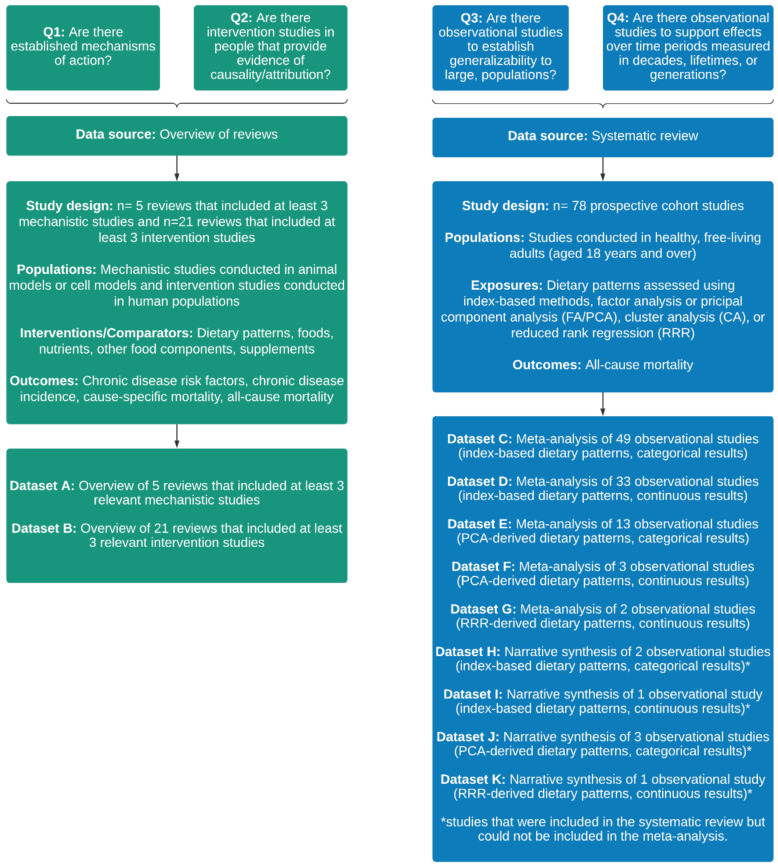
Overview of the data sources and datasets that were used in the application of the Hierarchies of Evidence Applied to Lifestyle Medicine (HEALM) approach.

An overview of the data sources and datasets that were used in the application of HEALM is provided in Figure 1. The datasets are described in more detail below and are available in the online Supplementary Materials.

### 3.1. Systematic Review

A total of 78 prospective cohort studies were included in the systematic review (Table S3). Data were collated and summarised for each of the five meta-analyses (Datasets C, D, E, F, and G) and the four narrative syntheses (Datasets H, I, J and K) that were conducted. Within each dataset, the number of studies with >1000 participants at follow-up ranged from one to 46, and the number of studies with length of follow-up >10 years ranged from one to 31. None of the included studies had low ROB. In one out of five meta-analyses, I2 was <50%. Small differences in effect size between studies were observed in two meta-analyses and one narrative synthesis. Overlap in CIs between studies was observed in all meta-analyses and none of the narrative syntheses. Within each dataset, the percentage of significant results ranged from 0 to 100.

### 3.2. Overview of Reviews

A total of 632 records were identified via hand searching and 100 duplicates were removed. Title and abstracts of the remaining 532 records were screened. Of these, 493 records were excluded and 39 progressed to full text screening. At the full text screening stage, 16 records were excluded based on study design and two records were excluded based on interventions/comparators. A total of 21 reviews were included in the overview of reviews (Table S4).

#### 3.2.1. Reviews That Included Mechanistic Studies

Five narrative reviews that included at least three relevant mechanistic studies were identified (Dataset A). All five reviews also included at least three relevant intervention studies. The number of mechanistic studies included in each review ranged from three to 62. Studies were conducted in animal models and in cell models. The interventions and comparators that were examined were either nutrients (e.g., fatty acids, Vitamin C) or other food components (e.g., lycopene, polyphenols). The outcomes that were examined included modulation of chronic inflammation and cancer cell proliferation or inhibition.

The ROB associated with included studies was not assessed by review authors, which meant that quality could not be assessed. Effect sizes and/or confidence intervals were not reported, which meant that consistency could not be assessed. Similarly, *p*-values and/or confidence intervals were not reported, which meant that significance could not be assessed.

#### 3.2.2. Reviews That Included Intervention Studies

A total of 21 reviews (14 narrative reviews and 7 systematic reviews) that included at least three relevant intervention studies were identified (Dataset B). The number of intervention studies included in each review ranged from three to 44. Studies were conducted in humans, including adults and children with and without disease. A broad range of interventions and comparators were examined, including dietary patterns (e.g., Mediterranean diet, Dietary Approaches to Stop Hypertension (DASH) diet), foods (e.g., fruits, vegetables) nutrients (e.g., fatty acids, carbohydrates), other food components (e.g., polyphenols, phytosterols) and supplements (e.g., Vitamin A, fish oil). Outcomes included chronic disease risk factors (e.g., blood pressure, blood lipids), chronic disease incidence (e.g., CVD, cancer), cause-specific mortality (e.g., CVD mortality, cancer mortality) and all-cause mortality. In one review (4.8%), most of the included studies had low ROB. There was an indication of consistent results between studies for at least one outcome in four reviews (19.0%). Most of the results were significant for at least one outcome in seven reviews (33.3%).

### 3.3. Strength of Evidence Assessed Using the HEALM Approach

The overall strength of evidence assessed using HEALM and comparison to the overall certainty of evidence assessed using GRADE is presented in Table 3. Further detail on the strength of evidence assessed using HEALM is provided in Table S5. Further detail on the certainty of evidence assessed using GRADE is reported elsewhere [30].

In the application of HEALM, the same dataset was used to answer Q1 and Q2, so in each row of Table 3, the response to Q1 and Q2 was the same. The response to Q1 was ‘uncertain’, because the evidence from mechanistic studies met the criteria for quantity, but not for quality, consistency, or significance. The response to Q2 was ‘yes’, because the evidence from intervention studies met the criteria for quantity, quality, consistency, and significance.

In each row of Table 3, the response to Q3 and Q4 was ‘uncertain’ or ‘no’. The response was ‘uncertain’ when the criteria for quantity were met, but the criteria for quality, quantity, and/or significance were not met. The response was ‘no’ when the criteria for quantity were not met, on the basis that quality, consistency, and significance do not matter if there is insufficient evidence (in terms of quantity) to answer the question.

In each row of Table 3, the overall strength of evidence was rated ‘Grade B: moderate/suggestive’ or ‘Grade C: insufficient/inconclusive’. In combination with the responses to Q1 and Q2, the evidence was rated ‘Grade B: moderate/suggestive’ when the response to Q3 and/or Q4 was ‘uncertain’, or ‘Grade C: insufficient/inconclusive’ when the response to Q3 and Q4 was ‘no’.

Using GRADE, the overall certainty of evidence was rated ‘Moderate’ (due to ‘serious’ risk of bias), ‘Low’ (due to ‘serious’ risk of bias and either ‘serious’ inconsistency or ‘serious’ imprecision’), or ‘Very low’ (due to ‘serious’ risk of bias and ‘very serious’ inconsistency).

## 4. Discussion

The aim of this study was to investigate how the HEALM approach could be used to assess the strength of evidence on associations between dietary patterns and all-cause mortality. Data from a systematic review and an overview of reviews were collected and summarised. Four criteria (quantity, quality, consistency, and significance) were developed and used in the application of HEALM. Using different datasets in combination, the overall strength of evidence was rated as ‘Grade B: moderate/suggestive’ or ‘Grade C: insufficient/inconclusive’.

The four criteria that were developed and used in the application of HEALM performed well when answering Q3 (evidence from large prospective cohort studies) and Q4 (evidence from long-term observational studies) using data from the systematic review. Each dataset included studies that were similar in terms of study design, population, intervention/comparator, and outcome, and from the outset, all included studies were considered relevant. This meant that in combination with study-level data on number of participants and length of follow-up, ‘quantity’ was easy to assess. The ROB associated with each included study had already been assessed, making it straightforward to assess ‘quality’. Within each dataset, result-level data were collated and summarised in a way that allowed ‘consistency’ and ‘significance’ to be assessed.

In contrast, these four criteria performed less well when answering Q1 (evidence from mechanistic studies in animal and cell models) and Q2 (evidence from intervention studies in humans) using data from the overview of reviews. The majority of included reviews were narrative reviews (n = 14, 66.7%). Narrative reviews tended to include studies that were diverse in terms of study design as well as the populations, interventions/comparators and outcomes examined. This made it difficult to identify the number of relevant mechanistic and intervention studies included in each review. The ROB associated with studies included in each narrative review was not assessed, which had implications for how ‘quality’ was assessed. Although the results of relevant mechanistic and intervention studies were described by review authors, effect sizes, CIs and/or *p*-values were usually not reported. This had implications for how ‘consistency’ and ‘significance’ were assessed. In the future, a more robust search strategy could be used to identify existing systematic reviews of mechanistic studies and intervention studies. For example, a search strategy that utilizes relevant databases and is developed in consultation with a librarian would be more robust. In the absence of existing systematic reviews, conducting a review of primary studies (rather than an overview of reviews) is advisable.

HEALM and GRADE are conceptually different in that the HEALM is used to assess evidence from mechanistic, intervention, and observational studies, whereas GRADE is used to assess evidence from either randomised or non-randomised studies [23,28]. This meant that different datasets were used in the application of HEALM and GRADE, which impacted the comparability of the overall ratings. However, results of this study indicate that regardless of the method used to assess the strength or certainty of evidence, the ROB associated with observational studies was a limiting factor in achieving the highest possible overall rating (‘Grade A: strong/decisive’ using HEALM or ‘high’ certainty using GRADE). The ROB associated with each prospective cohort study included in the systematic review was assessed as ‘low’ or ‘moderate’ using ROBINS-I. Using HEALM, this meant that the response to Q3 and Q4 was ‘uncertain’ at best. The implication was that even in the presence of intervention studies in people that provide evidence of causality/attribution (the response to Q2 was ‘yes’), the overall strength of the evidence was rated as ‘Grade B: moderate/suggestive’ at best. Similarly, using GRADE, the evidence was downgraded based on ‘risk of bias’, which meant that the overall certainty of evidence was ‘moderate’ at best. Going forward, careful consideration should be given to the method used to assess ROB associated with individual studies. For example, use of nutrition-specific tools such as the Risk of Bias for Nutrition Observational Studies (ROB-Nobs) Tool [4] and the Nutrition Quality Evaluation Strengthening Tools (NUQUEST) [36] may be more suitable than ROBINS-I. Further research is needed to investigate the impact of using nutrition-specific ROB tools on the ‘strength’, ‘certainty’ or ‘quality’ of evidence assessed using HEALM, GRADE, and NutriGrade [37,38].

In the context of dietary guideline development, HEALM is promising in that it provides a new approach for integrating and assessing the overall strength of evidence from mechanistic, intervention, and observational studies, which is important because evidence from observational studies alone is insufficient to infer causality [7,8]. However, results of this study suggest that in order to apply HEALM, data from *systematic* reviews of relevant evidence from mechanistic studies, intervention studies, and observational studies are needed. In the absence of existing systematic reviews, new reviews may need to be conducted. Conducting new systematic reviews is resource-intensive [39,40]. Ideally, for research questions that are commonly asked as part of the dietary guideline development process, systematic reviews that synthesise relevant evidence from mechanistic studies, intervention studies, and observational studies would be conducted and continually updated by nutrition researchers. For each research question, the overall strength of the evidence could then be assessed by using HEALM (using data from various combinations of existing systematic reviews).

Inconsistent terminology presents another challenge for researchers and dietary guideline developers seeking to integrate and assess the overall strength of evidence from mechanistic, intervention, and observational studies. For example, using HEALM, mechanistic evidence is derived from studies conducted in animal and cell models, and evidence that can be used to demonstrate causation is derived from intervention studies conducted in humans [28]. In contrast, using the WCRF approach, evidence from experimental studies conducted in humans or in animals can be used to demonstrate biological plausibility [7]. In the nutrition literature more broadly, evidence from RCTs (conducted in humans) is often described as mechanistic evidence that can be used in combination with evidence from observational studies to infer causation [12,13,17]. These inconsistencies in terminology may have implications for the methods used to conduct individual systematic reviews (e.g., selection of the most appropriate ROB tool for assessing individual studies) and the methods used to integrate and assess the strength of the evidence for the purpose of dietary guideline development.

This study provides a worked example of how the HEALM approach can be used to assess the strength of evidence for dietary exposures and health outcomes. The criteria that were used in the application of HEALM were developed based on the information provided by the creators of HEALM, but the interpretation of this information, and the decisions that were made in how the criteria were used to answer Q1–4 were subjective. Each of these methodological decisions had implications for how the overall strength of evidence was rated. However, the study builds on previous work to demonstrate how HEALM can be applied in the field of nutrition. It has highlighted some of the complexities associated with the integration and assessment of evidence from mechanistic, intervention, and observational studies for research questions that are difficult to address using evidence from RCTs. Robust quality assessment methods are needed to ensure relevant, high-quality evidence is translated into dietary guidelines, and this study make a valuable contribution to the literature by testing the application of a novel method using a rigorous and transparent approach.

## 5. Conclusions

Dietary guidelines should be informed by relevant, high-quality evidence on associations between diet and health. This study investigated how the HEALM approach could be used to assess the strength of evidence on associations between dietary patterns and all-cause mortality. A set of four criteria were developed and used in the application of HEALM. The overall strength of evidence was rated ‘Grade B: moderate/suggestive’ or ‘Grade C: insufficient/inconclusive’. HEALM provides a new approach for integrating and assessing the overall strength of evidence from mechanistic, intervention, and observational studies, but some practical challenges associated with the application of HEALM were identified. Further research is needed to address these challenges, and to investigate the use of nutrition-specific ROB tools in combination with quality assessment methods, including HEALM and GRADE.

## Figures and Tables

**Table 1:** Overview of the Hierarchies of Evidence Applied to Lifestyle Medicine (HEALM) approach ^a^.

Q1: Are there established mechanisms of action? A plurality ^b^ of evidence from bench science and animal models.	Q2: Are there intervention studies in people that provide evidence of causality/attribution? A plurality ^b^ of high-quality intervention trials, randomized controlled trials, interim measures, and surrogate markers as outcomes.	Q3: Are there observational studies to establish generalizability to large, populations? A plurality ^b^ of high-quality evidence from large prospective cohort studies.	Q4: Are there observational studies to support effects over time periods measured in decades, lifetimes, or generations? A plurality ^b^ of evidence from high quality, long-term observational studies; retrospective cohort studies; ethnography; transcultural studies.	Overall strength of evidence
Response ^c^ Yes = 2 Uncertain = 1 No = 0	Response ^c^ Yes = 3 Uncertain = 1 No = 0	Response ^c^ Yes = 2 Uncertain = 1 No = 0	Response ^c^ Yes = 2 Uncertain = 1 No = 0	Overall score 0–9 Overall rating ^d^ Grade A: strong/decisive (overall score ≥ 7) Grade B: moderate/suggestive (overall score 5 or 6) Grade C: insufficient/inconclusive (overall score < 5)

^a^ Adapted from Katz et al. 2019 [28]. ^b^ “Plurality may vary depending on the total number of existing studies conducted on a particular research question and must be determined on a case-by-case basis. For example, three consistent studies from a variety of study design with no opposing studies may constitute a plurality. Were there to be opposing studies the target number would be more than three” [28] (p. 12). ^c^ “Answers to scoring questions should be based on expert consensus in evaluating available evidence. Evidence is conclusive when it can be identified as sufficient in quantity and quality, and consistent in findings, fostering clear consensus among experts. This would generally mean a replicated finding, and consistent effects among a clear plurality of high quality, related publications. Evidence is uncertain when studies are few, small, poor quality, or conflicting but generally suggestive of a particular finding” [28] (p. 12). “While expert consensus is critical in evaluation, a framework to inform discussion based on quantitative criteria used in previous umbrella reviews is suggested: 1. Total sample and number of cases of included studies. 2. Significance of association based on *p*-values (highly significant defined as *p* < 0.0001 vs. nominally significant defined as *p* < 0.05) and confidence intervals that exclude vs. include the null value. 3. When considering studies that include meta-analyses, a target threshold of 1000 cases, no evidence of small-study effects or excess significance bias, a 95% prediction interval excluding the null value and no large, unexplained, between-study heterogeneity (I2 < 50%)” [28] (p. 12). ^d^ “Grade A: Strong/decisive = ≥7. This would require decisive evidence in all other categories, AND at least suggestive evidence from intervention trials in people; OR strong evidence from intervention trials in people, and decisive evidence in other two categories; OR strong evidence from intervention trials, decisive evidence in any other category, and suggestive evidence in the remaining two. Lends a primacy to RCT evidence but allows for strong evidence even with nothing more than suggestive evidence in intervention trial category. Grade B: Moderate/suggestive = 5 or 6. Achievable with decisive intervention trial evidence, and strong evidence in ANY other category. OR, strong evidence in all categories other than intervention trials. Grade C: Insufficient/inconclusive = < 5” [28] (p. 12).

**Table 2:** Overview of the criteria developed and used in the application of the Hierarchies of Evidence Applied to Lifestyle Medicine (HEALM) approach ^a^.

Q1: Are there established mechanisms of action?	Q2: Are there intervention studies in people that provide evidence of causality/attribution?	Q3: Are there observational studies to establish generalizability to large, populations?	Q4: Are there observational studies to support effects over time periods measured in decades, lifetimes, or generations?	Overall strength of evidence
Data source: overview of reviews	Data source: overview of reviews	Data source: systematic review	Data source: systematic review	Data source: overview of reviews + systematic review
Quantity ^b^ Is there evidence from mechanistic studies? Yes if at least 1 review included at least 3 mechanistic studies. No if no reviews included at least 3 mechanistic studies.	Quantity ^b^ Is there evidence from intervention studies? Yes if at least 1 review included at least 3 intervention studies. No if no reviews included at least 3 intervention studies.	Quantity ^b^ Is there evidence from large, prospective cohort studies? Yes if evidence was derived from at least 3 prospective cohort studies that included >1000 participants at follow up. No if evidence was derived from <3 prospective cohort studies that included >1000 participants at follow up.	Quantity ^b^ Is there evidence from observational studies conducted over time periods measured in decades, lifetimes, or generations? Yes if evidence was derived from at least 3 studies with length of follow-up >10 years. No if evidence was derived from <3 studies with length of follow-up >10 years.	
Quality ^b^ Was most of the evidence derived from studies with low ROB? Yes if most of the evidence was derived from studies with low ROB in at least 1 review. No if most of the evidence was derived from studies with low ROB in <1 review.	Quality ^b^ Was most of the evidence derived from studies with low ROB? Yes if most of the evidence was derived from studies with low ROB in at least 1 review. No if most of the evidence was derived from studies with low ROB in <1 review.	Quality ^b^ Was most of the evidence derived from studies with low ROB? Yes if >50% of studies had low ROB. No if ≤50% of studies had low ROB.	Quality ^b^ Was most of the evidence derived from studies with low ROB? Yes if >50% of studies had low ROB. No if ≤50% of studies had low ROB.	
Consistency ^b^ Is there an indication of consistent results between studies? Yes if there was an indication of consistent results between studies for at least 1 outcome in at least 1 review. No if there was an indication of consistent results between studies in <1 review.	Consistency ^b^ Is there an indication of consistent results between studies? Yes if there was an indication of consistent results between studies for at least 1 outcome in at least 1 review. No if there was an indication of consistent results between studies in <1 review.	Consistency ^b^ Is there an indication of consistent results between studies? Yes if I2 < 50% and/or small differences in effect size and/or overlap in Cis between studies. No if I2 ≥ 50% and/or large differences in effect size and/or NO overlap in CIs between studies.	Consistency ^b^ Is there an indication of consistent results between studies? Yes if I2 < 50% and/or small differences in effect size and/or overlap in CIs between studies. No if I2 ≥ 50% and/or large differences in effect size and/or NO overlap in CIs between studies.	
Significance ^b^ Were most of the results significant? Yes if most of the results were significant for at least 1 outcome in at least 1 review. No if most of the results were significant in <1 review.	Significance ^b^ Were most of the results significant? Yes if most of the results were significant for at least 1 outcome in at least 1 review. No if most of the results were significant in <1 review.	Significance ^b^ Were most of the results significant? Yes if >50% of results (not studies) were significant (*p*-value <0.05 and/or CI does NOT cross 1). No if ≤ 50% of results (not studies) were significant.	Significance ^b^ Were most of the results significant? Yes if >50% of results (not studies) were significant (*p*-value < 0.05 and/or CI does NOT cross 1). No if ≤50% of results (not studies) were significant.	
Response Yes = 2 Uncertain = 1 No = 0	Response Yes = 3 Uncertain = 1 No = 0	Response Yes = 2 Uncertain = 1 No = 0	Response Yes = 2 Uncertain = 1 No = 0	Overall score 0–9 Overall rating Grade A: strong/decisive (overall score ≥ 7) Grade B: moderate/suggestive (overall score 5 or 6) Grade C: insufficient/inconclusive (overall score < 5)

CI confidence interval; ROB risk of bias. ^a^ Adapted from Katz et al. [28]. ^b^ Criteria developed by the authors of this study using information from multiple sources [23,28,35].

**Table 3:** Overall strength of evidence assessed using the Hierarchies of Evidence Applied to Lifestyle Medicine (HEALM) approach and comparison to overall certainty of evidence assessing using the Grading of Recommendations, Assessment, Development and Evaluation (GRADE) approach ^a^.

Q1: Are there established mechanisms of action?	Q2: Are there intervention studies in people that provide evidence of causality/attribution?	Q3: Are there observational studies to establish generalizability to large, populations?	Q4: Are there observational studies to support effects over time periods measured in decades, lifetimes, or generations?	Overall strength of evidence assessed using HEALM	Overall certainty of evidence assessed using GRADE
Dataset A	Dataset B	Dataset C	Dataset C	Dataset A + B + C	Dataset C
Response Yes = 2 **Uncertain = 1** No = 0 *‘Yes’ for quantity, but ‘no’ for quality, consistency, and significance.*	Response **Yes = 3** Uncertain = 1 No = 0 *‘Yes’ for quantity, quality, consistency, and significance.*	Response Yes = 2 **Uncertain = 1** No = 0 *‘Yes’ for quantity and significance and ‘no’ for quality and consistency.*	Response Yes = 2 **Uncertain = 1** No = 0 *‘Yes’ for quantity and significance and ‘no’ for quality and consistency.*	Overall score 6 Overall rating Grade A: strong/decisive (overall score ≥ 7) **Grade B: moderate/suggestive (overall score 5 or 6)** Grade C: insufficient/inconclusive (overall score < 5)	Overall rating High Moderate Low **Very low** *Downgraded due to ‘serious’ risk of bias and ‘very serious’ inconsistency.*
Dataset A	Dataset B	Dataset D	Dataset D	Dataset A + B + D	Dataset D
Response Yes = 2 **Uncertain = 1 ** No = 0 *‘Yes’ for quantity, but ‘no’ for quality, consistency, and significance.*	Response **Yes = 3** Uncertain = 1 No = 0 *‘Yes’ for quantity, quality, consistency, and significance.*	Response Yes = 2 **Uncertain = 1** No = 0 *‘Yes’ for quantity and significance and ‘no’ for quality and consistency.*	Response Yes = 2 **Uncertain = 1** No = 0 *‘Yes’ for quantity and significance and ‘no’ for quality and consistency.*	Overall score 6 Overall rating Grade A: strong/decisive (overall score ≥ 7) **Grade B: moderate/suggestive (overall score 5 or 6)** Grade C: insufficient/inconclusive (overall score < 5)	Overall rating High Moderate **Low** Very low *Downgraded due to ‘serious’ risk of bias and ‘serious’ inconsistency.*
Dataset A	Dataset B	Dataset E	Dataset E	Dataset A + B + E	Dataset E
Response Yes = 2 **Uncertain = 1 ** No = 0 *‘Yes’ for quantity, but ‘no’ for quality, consistency, and significance.*	Response **Yes = 3** Uncertain = 1 No = 0 *‘Yes’ for quantity, quality, consistency, and significance.*	Response Yes = 2 **Uncertain = 1** No = 0 *‘Yes’ for quantity and significance and ‘no’ for quality and consistency.*	Response Yes = 2 **Uncertain = 1** No = 0 *‘Yes’ for quantity and significance and ‘no’ for quality and consistency.*	Overall score 6 Overall rating Grade A: strong/decisive (overall score ≥ 7) **Grade B: moderate/suggestive (overall score 5 or 6)** Grade C: insufficient/inconclusive (overall score < 5)	Overall rating High Moderate **Low** Very low *Downgraded due to ‘serious’ risk of bias and ‘serious’ inconsistency.*
Dataset A	Dataset B	Dataset F	Dataset F	Dataset A + B + F	Dataset F
Response Yes = 2 **Uncertain = 1 ** No = 0 *‘Yes’ for quantity, but ‘no’ for quality, consistency, and significance.*	Response **Yes = 3** Uncertain = 1 No = 0 *‘Yes’ for quantity, quality, consistency, and significance.*	Response Yes = 2 Uncertain = 1 **No = 0** *‘No’ for quantity. Quality, consistency, and significance don’t matter if there isn’t enough evidence.*	Response Yes = 2 Uncertain = 1 **No = 0** *‘No’ for quantity. Quality, consistency, and significance don’t matter if there isn’t enough evidence.*	Overall score 4 Overall rating Grade A: strong/decisive (overall score ≥ 7) Grade B: moderate/suggestive (overall score 5 or 6) **Grade C: insufficient/inconclusive (overall score < 5)**	Overall rating High Moderate **Low** Very low *Downgraded due to ‘serious’ risk of bias and ‘serious’ inconsistency.*
Dataset A	Dataset B	Dataset G	Dataset G	Dataset A + B + G	Dataset G
Response Yes = 2 **Uncertain = 1 ** No = 0 *‘Yes’ for quantity, but ‘no’ for quality, consistency, and significance.*	Response **Yes = 3** Uncertain = 1 No = 0 *‘Yes’ for quantity, quality, consistency, and significance.*	Response Yes = 2 Uncertain = 1 **No = 0** *‘No’ for quantity. Quality, consistency, and significance don’t matter if there isn’t enough evidence.*	Response Yes = 2 Uncertain = 1 **No = 0** *‘No’ for quantity. Quality, consistency, and significance don’t matter if there isn’t enough evidence.*	Overall score 4 Overall rating Grade A: strong/decisive (overall score ≥ 7) Grade B: moderate/suggestive (overall score 5 or 6) **Grade C: insufficient/inconclusive (overall score < 5)**	Overall rating High **Moderate** Low Very low *Downgraded due to ‘serious’ risk of bias.*
Dataset A	Dataset B	Dataset H	Dataset H	Dataset A + B + H	Dataset H
Response Yes = 2 **Uncertain = 1 ** No = 0 *‘Yes’ for quantity, but ‘no’ for quality, consistency, and significance.*	Response **Yes = 3** Uncertain = 1 No = 0 *‘Yes’ for quantity, quality, consistency, and significance.*	Response Yes = 2 Uncertain = 1 **No = 0** *‘No’ for quantity. Quality, consistency, and significance don’t matter if there isn’t enough evidence.*	Response Yes = 2 Uncertain = 1 **No = 0** *‘No’ for quantity. Quality, consistency, and significance don’t matter if there isn’t enough evidence.*	Overall score 4 Overall rating Grade A: strong/decisive (overall score ≥ 7) Grade B: moderate/suggestive (overall score 5 or 6) **Grade C: insufficient/inconclusive (overall score < 5)**	Overall rating High Moderate **Low** Very low *Downgraded due to ‘serious’ risk of bias and ‘serious’ inconsistency.*
Dataset A	Dataset B	Dataset I	Dataset I	Dataset A + B + I	Dataset I
Response Yes = 2 **Uncertain = 1 ** No = 0 *‘Yes’ for quantity, but ‘no’ for quality, consistency, and significance.*	Response **Yes = 3** Uncertain = 1 No = 0 *‘Yes’ for quantity, quality, consistency, and significance.*	Response Yes = 2 Uncertain = 1 **No = 0** *‘No’ for quantity. Quality, consistency, and significance don’t matter if there isn’t enough evidence.*	Response Yes = 2 Uncertain = 1 **No = 0** *‘No’ for quantity. Quality, consistency, and significance don’t matter if there isn’t enough evidence.*	Overall score 4 Overall rating Grade A: strong/decisive (overall score ≥ 7) Grade B: moderate/suggestive (overall score 5 or 6) **Grade C: insufficient/inconclusive (overall score < 5)**	Overall rating High **Moderate** Low Very low *Downgraded due to ‘serious’ risk of bias.*
Dataset A	Dataset B	Dataset J	Dataset J	Dataset A + B + J	Dataset J
Response Yes = 2 **Uncertain = 1 ** No = 0 *‘Yes’ for quantity, but ‘no’ for quality, consistency, and significance.*	Response **Yes = 3** Uncertain = 1 No = 0 *‘Yes’ for quantity, quality, consistency, and significance.*	Response Yes = 2 **Uncertain = 1** No = 0 *‘Yes’ for quantity and consistency, and ‘no’ for quality and significance.*	Response Yes = 2 Uncertain = 1 **No = 0** *‘No’ for quantity. Quality, consistency, and significance don’t matter if there isn’t enough evidence.*	Overall score 5 Overall rating Grade A: strong/decisive (overall score ≥ 7) **Grade B: moderate/suggestive (overall score 5 or 6)** Grade C: insufficient/inconclusive (overall score < 5)	Overall rating High Moderate **Low** Very low *Downgraded due to ‘serious’ risk of bias and ‘serious’ imprecision.*
Dataset A	Dataset B	Dataset K	Dataset K	Dataset A + B + K	Dataset K
Response Yes = 2 **Uncertain = 1 ** No = 0 *‘Yes’ for quantity, but ‘no’ for quality, consistency, and significance.*	Response **Yes = 3** Uncertain = 1 No = 0 *‘Yes’ for quantity, quality, consistency, and significance.*	Response Yes = 2 Uncertain = 1 **No = 0** *‘No’ for quantity. Quality, consistency, and significance don’t matter if there isn’t enough evidence.*	Response Yes = 2 Uncertain = 1 **No = 0** *‘No’ for quantity. Quality, consistency, and significance don’t matter if there isn’t enough evidence.*	Overall score 4 Overall rating Grade A: strong/decisive (overall score ≥ 7) Grade B: moderate/suggestive (overall score 5 or 6) **Grade C: insufficient/inconclusive (overall score < 5)**	Overall rating High Moderate **Low** Very low *Downgraded due to ‘serious’ risk of bias and ‘serious’ imprecision.*

CI confidence intervals; MA meta-analysis; NS narrative synthesis; PCA principal component analysis; ROB risk of bias; RRR reduced rank regression. Dataset A: Overview of 5 reviews that included at least 3 relevant mechanistic studies. Dataset B: Overview of 21 reviews that included at least 3 relevant intervention studies. Dataset C: MA of 49 observational studies (index-based dietary patterns, categorical results). Dataset D: MA of 33 observational studies (index-based dietary patterns, continuous results). Dataset E: MA of 13 observational studies (PCA-derived dietary patterns, categorical results). Dataset F: MA of 3 observational studies (PCA-derived dietary patterns, continuous results). Dataset G: MA of 2 observational studies (RRR-derived dietary patterns, continuous results). Dataset H: NS of 2 observational studies (index-based dietary patterns, categorical results) ^b^. Dataset I: NS of 1 observational study (index-based dietary patterns, continuous results) ^b^. Dataset J: NS of 3 observational studies (PCA-derived dietary patterns, categorical results) ^b^. Dataset K: NS of 1 observational study (RRR-derived dietary patterns, continuous results ^b^. ^a^ Selected responses indicated in bold. Comments explaining selected responses indicated in italics. ^b^ Studies that were included in the systematic review but could not be included in the MA.

## Data Availability

The data used in this study are available in the online Supplementary Materials (Datasets A–K).

## Supplementary Materials

The following supporting information can be downloaded at: https://www.mdpi.com/article/10.3390/nu14204340/s1, Table S1: Eligibility criteria for the systematic review and the overview of reviews; Figure S1: Search strategy for the overview of reviews; Table S2: Data items for the systematic review and the overview of reviews; Table S3: Studies included in the systematic review of dietary patterns and all-cause mortality (n = 78); Table S4: Reviews included in the overview of reviews (n = 21); Table S5: Strength of evidence assessed using the Hierarchies of Evidence Applied to Lifestyle Medicine (HEALM) approach; Datasets A–K.
