# Supplementary material for: Using the Hierarchies of Evidence Applied to Lifestyle Medicine (HEALM) Approach to Assess the Strength of Evidence on Associations between Dietary Patterns and All-Cause Mortality
Source: Nutrients. 2022 Oct 17;14(20):4340. doi: 10.3390/nu14204340 (PMC9609205; doi:10.3390/nu14204340)
Supplement: Supplementary file 1 [file nutrients-14-04340-s001.zip › nutrients-1941976 supplementary material_revised 13.10.22.pdf]

nutrients

MDPI

# Supplementary material

**Contents**

**Table S1.** Eligibility criteria for the systematic review and the overview of reviews. ........ 2

**Figure S1.** Search strategy for the overview of reviews. ........ 5

**Table S2.** Data items for the systematic review and the overview of reviews. ........ 6

**Table S3.** Studies included in the systematic review of dietary patterns and all-cause mortality (n=78). ........ 9

**Table S4.** Reviews included in the overview of reviews (n=21). ........ 15

**Table S5.** Strength of evidence assessed using the Hierarchies of Evidence Applied to Lifestyle Medicine (HEALM) approach ........ 17

**Table S1.** Eligibility criteria for the systematic review and the overview of reviews.

| **Data source** | **Systematic review** | | **Overview of reviews** | |
|---|---|---|---|---|
| **Criteria** | **Include** | **Exclude** | **Include** | **Exclude** |
| Publication details | Studies published in any language between January 1980 and March 2019. | Studies published prior to 1980. | Reviews published in English prior to March 2019. | Reviews not published in Englished or full text not available. |
| Study designs | Randomised controlled trials, prospective cohort studies, and nested case control studies. | Primary studies with any other design (e.g. cross-sectional studies, ecological studies).<br><br>Reviews, conference proceedings, conference abstracts, study protocols, commentaries, editorials, reports, books. | Reviews that include 3 or more relevant mechanistic studies.<br><br>Reviews that include 3 or more relevant intervention studies. | Reviews that include less than 3 relevant mechanistic studies.<br><br>Reviews that include less than 3 relevant intervention studies.<br><br>Reviews that only include observational studies.<br><br>Reviews that only include other reviews.<br><br>Primary studies, conference proceedings, conference abstracts, study protocols, commentaries, editorials, reports, books. |

| | | | | |
|---|---|---|---|---|
| Populations | Studies conducted in healthy, free-living adults (aged 18 years and over). | Studies conducted exclusively in specialist populations e.g. institutionalised populations, diseased populations, pregnant women.<br><br>Studies conducted in animals. | For mechanistic studies: studies conducted in animal models or cell models.<br><br>For intervention studies: studies conducted in any human population (including e.g. adults and children with and without disease). | For mechanistic studies: studies conducted in humans.<br><br>For intervention studies: studies conducted in animal models or cell models. |
| Interventions/Comparators | For prospective cohort and nested case control studies: dietary pattern exposures assessed using index-based methods, factor or principal component analysis, cluster analysis, or reduced rank regression.<br><br>For randomised controlled trials: studies were also eligible for inclusion if two or more food groups were consumed together as part of a dietary pattern. | Other diet exposures e.g. foods, nutrients, supplements (not assessed as part of a dietary pattern).<br><br>Non-diet exposures e.g. physical activity.<br><br>Combined exposures e.g. diet combined with exercise, physical activity, sedentary behaviour, smoking, sleep, drugs. | Dietary patterns, foods, nutrients, other food components, supplements. | Non-diet exposures e.g. physical activity.<br><br>Combined exposures e.g. diet combined with exercise, physical activity, sedentary behaviour, smoking, sleep, drugs. |
| Outcomes | All-cause mortality. | Cause-specific mortality, any other health outcome. | Any health outcome e.g. chronic disease risk factors, | Non-health outcomes e.g. environmental outcomes |

| | | | | |
|---|---|---|---|---|
| | | | chronic disease incidence, cause-specific and all-cause mortality. | associated with food production or consumption. |

**Figure S1.** Search strategy for the overview of reviews.

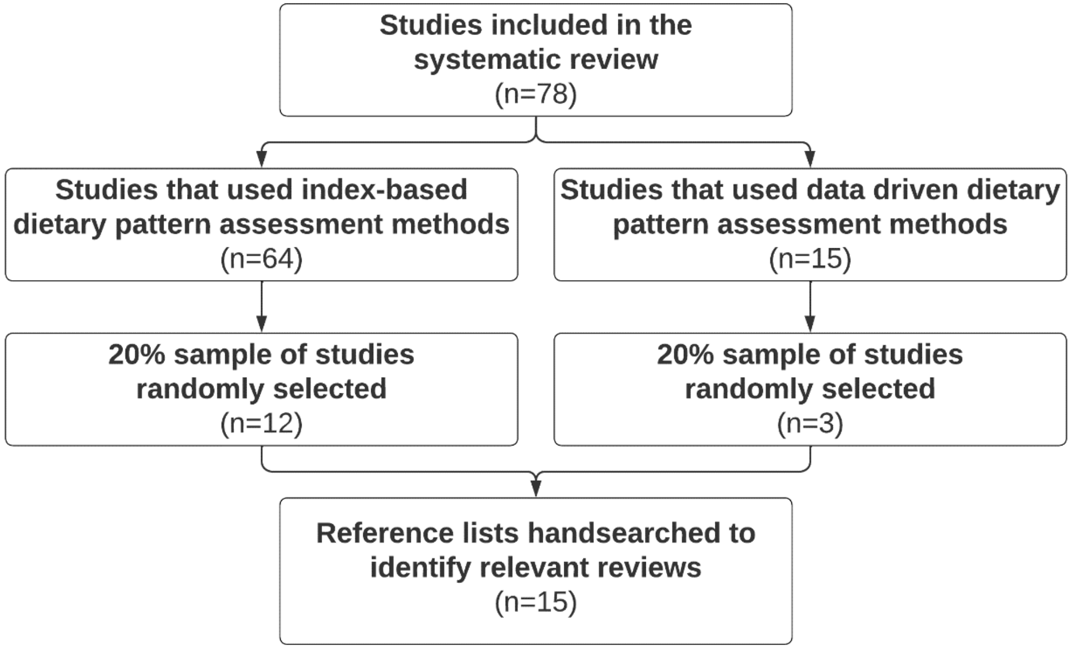

**Table S2.** Data items for the systematic review and the overview of reviews.

| **Systematic review** | **Overview of reviews** |
|---|---|
| The following data were collated from the studies included in each meta-analysis and each narrative synthesis:<br>- For each study: number of participants at follow-up; length of follow-up; overall ROB (low, moderate, serious, or critical)<br>- For each result: Relative measure of effect size (e.g. Relative Risk, Hazard Ratio, Odds Ratio), 95% confidence intervals (CIs) for relative measure of effect size | The following data were extracted from each review:<br>- Review type (systematic review or narrative review)<br>- Number of relevant mechanistic studies<br>o For each study: populations, interventions/comparators, and outcomes<br>o Was most of the evidence derived from studies with low ROB?<br>▪ Yes if >50% of studies had low ROB<br>▪ No if <=50% of studies had low ROB<br>o Is there an indication of consistent results between studies for at least 1 outcome?<br>▪ Yes if I2<50% and/or small differences in effect size and/or overlap in CIs between studies<br>▪ No if I2>=50% and/or large differences in effect size and/or NO overlap in CIs between studies<br>o Were most of the results significant for at least 1 outcome?<br>▪ Yes if >50% of results (not studies) were significant (p-value <0.05 and/or CI does NOT cross 1 for relative measures of effect and/or CI does NOT cross 0 for mean difference in effect)<br>▪ No if <=50% of results (not studies) were significant |

| | |
|---|---|
| | - Number of relevant intervention studies<br>o For each study: populations, interventions/comparators, and outcomes<br>o Was most of the evidence derived from studies with low ROB? (yes/no)<br>▪ Yes if >50% of studies had low ROB<br>▪ No if <=50% of studies had low ROB<br>o Is there an indication of consistent results between studies for at least 1 outcome?<br>▪ Yes if I2<50% and/or small differences in effect size and/or overlap in CIs between studies<br>▪ No if I2>=50% and/or large differences in effect size and/or NO overlap in CIs between studies<br>o Were most of the results significant for at least 1 outcome?<br>▪ Yes if >50% of results (not studies) were significant (p-value <0.05 and/or CI does NOT cross 1 for relative measures of effect and/or CI does NOT cross 0 for mean difference in effect)<br>▪ No if <=50% of results (not studies) were significant |
| The data were summarised as follows:<br>- Number of prospective cohort studies with >1000 participants at follow-up | The data were summarised as follows:<br>- Number of reviews that included at least 3 mechanistic studies |

| | |
|---|---|
| - Number of prospective cohort studies with length of follow-up >10 years<br>- Percentage of studies with low ROB<br>- I2<50% (yes/no)<br>- Small differences in effect size between studies (yes/no)<br>- Overlap in CIs between studies (yes/no)<br>- Number and percentage of significant results | o Number and percentage of narrative reviews and systematic reviews<br>o Number and percentage of reviews where most studies had low ROB<br>o Number and percentage of reviews with an indication of consistent results between studies for at least one outcome<br>o Number and percentage of reviews where most results were significant for at least one outcome<br>- Number of reviews that included at least 3 intervention studies<br>o Number and percentage of narrative reviews and systematic reviews<br>o Number and percentage of reviews where most studies had low ROB<br>o Number and percentage of reviews with an indication of consistent results between studies for at least one outcome<br>o Number and percentage of reviews where most results were significant for at least one outcome |

CI confidence interval; ROB risk of bias

**Table S3.** Studies included in the systematic review of dietary patterns and all-cause mortality (n=78).

| ID[a] | Citation |
|---|---|
| 10 | Akbaraly TN, Ferrie JE, Berr C, et al. (2011) Alternative healthy eating index and mortality over 18 y of follow-up: Results from the Whitehall II cohort. Am J Clin Nutr. 94(1):247-53. http://doi.org/10.3945/ajcn.111.013128 |
| 21 | Alvarez-Alvarez I, Zazpe I, Pérez de Rojas J, et al. (2018) Mediterranean diet, physical activity and their combined effect on all-cause mortality: The Seguimiento Universidad de Navarra (SUN) cohort. Prev Med. 106:45-52. http://doi.org/10.1016/j.ypmed.2017.09.021 |
| 41 | Bamia C, Trichopoulos D, Ferrari P, et al. (2007) Dietary patterns and survival of older Europeans: The EPIC-Elderly Study (European Prospective Investigation into Cancer and Nutrition). Public Health Nutr. 10(6):590-8. http://doi.org/10.1017/S1368980007382487 |
| 45 | Behrens G, Fischer B, Kohler S, et al. (2013) Healthy lifestyle behaviors and decreased risk of mortality in a large prospective study of U.S. women and men. Eur J Epidemiol. 28(5):361-72. http://doi.org/10.1007/s10654-013-9796-9 |
| 47 | Bellavia A, Tektonidis TG, Orsini N, et al. (2016) Quantifying the benefits of Mediterranean diet in terms of survival. Eur J Epidemiol. 31(5):527-30. http://doi.org/10.1007/s10654-016-0127-9 |
| 60 | Biesbroek S, Verschuren WMM, Boer JMA, et al. (2017) Does a better adherence to dietary guidelines reduce mortality risk and environmental impact in the Dutch sub-cohort of the European Prospective Investigation into Cancer and Nutrition? Br J Nutr. 118(1):69-80. http://doi.org/10.1017/S0007114517001878 |
| 62 | Bonaccio M, Di Castelnuovo A, Costanzo S, et al. (2018) Mediterranean diet and mortality in the elderly: A prospective cohort study and a meta-analysis. Br J Nutr. http://doi.org/10.1017/S0007114518002179 |
| 64 | Bongard V, Arveiler D, Dallongeville J, et al. (2016) Food groups associated with a reduced risk of 15-year all-cause death. Eur J Clin Nutr. 70(6):715-22. http://doi.org/10.1038/ejcn.2016.19 |
| 68 | van den Brandt PA (2011) The impact of a Mediterranean diet and healthy lifestyle on premature mortality in men and women. Am J Clin Nutr. 94(3):913-20. http://doi.org/10.3945/ajcn.110.008250 |
| 71 | Buckland G, Agudo A, Travier N, et al. (2011) Adherence to the Mediterranean diet reduces mortality in the Spanish cohort of the European Prospective Investigation into Cancer and Nutrition (EPIC-Spain). Br J Nutr. 106(10):1581-91. http://doi.org/10.1017/S0007114511002078 |
| 85 | Cai H, Shu XO, Gao YT, et al. (2007) A prospective study of dietary patterns and mortality in Chinese women. Epidemiology. 18(3):393-401. http://doi.org/10.1097/01.ede.0000259967.21114.45 |
| 112 | Dai J, Krasnow RE, Reed T (2016) Midlife moderation-quantified healthy diet and 40-year mortality risk from CHD: The prospective National Heart, Lung, and Blood Institute Twin Study. Br J Nutr. 116(2):326-34. http://doi.org/10.1017/S0007114516001914 |

| | |
|---|---|
| 156 | Ford DW, Hartman TJ, Still C, et al. (2014) Body mass index, poor diet quality, and health-related quality of life are associated with mortality in rural older adults. J Nutr Gerontol Geriatr. 33(1):23-34. http://doi.org/10.1080/21551197.2014.875819 |
| 159 | Franzon K, Byberg L, Sjögren P, et al. (2017) Predictors of Independent Aging and Survival: A 16-Year Follow-Up Report in Octogenarian Men. J Am Geriatr Soc. 65(9):1953-60. http://doi.org/10.1111/jgs.14971 |
| 180 | George SM, Ballard-Barbash R, Manson JE, et al. (2014) Comparing indices of diet quality with chronic disease mortality risk in postmenopausal women in the women's health initiative observational study: evidence to inform national dietary guidance. Am J Epidemiol. 180(6):616-25. http://doi.org/10.1093/aje/kwu173 |
| 193 | Harmon BE, Boushey CJ, Shvetsov YB, et al. (2015) Associations of key diet-quality indexes with mortality in the Multiethnic Cohort: the Dietary Patterns Methods Project. Am J Clin Nutr. 101(3):587-97. http://doi.org/10.3945/ajcn.114.090688 |
| 200 | Heidemann C, Schulze MB, Franco OH, et al. (2008) Dietary patterns and risk of mortality from cardiovascular disease, cancer, and all causes in a prospective cohort of women. Circulation. 118(3):230-7. http://doi.org/10.1161/CIRCULATIONAHA.108.771881 |
| 208 | Hodge AM, Bassett JK, Dugué PA, et al. (2018) Dietary inflammatory index or Mediterranean diet score as risk factors for total and cardiovascular mortality. Nutr Metab Cardiovasc Dis. 28(5):461-9. http://doi.org/10.1016/j.numecd.2018.01.010 |
| 223 | Huijbregts P, Feskens E, Rasanen L, et al. (1997) Dietary pattern and 20 year mortality in elderly men in Finland, Italy, and The Netherlands: longitudinal cohort study. BMJ. 315(7099):13-7. http://doi.org/10.1136/bmj.315.7099.13 |
| 228 | Jacobs S, Harmon BE, Ollberding NJ, et al. (2016) Among 4 diet quality indexes, only the alternate mediterranean diet score is associated with better colorectal cancer survival and only in African American women in the multiethnic Cohort. J Nutr. 146(9):1746-55. http://doi.org/10.3945/jn.116.234237 |
| 237 | Jones NRV, Forouhi NG, Khaw KT, et al. (2018) Accordance to the Dietary Approaches to Stop Hypertension diet pattern and cardiovascular disease in a British, population-based cohort. Eur J Epidemiol. 33(2):235-44. http://doi.org/10.1007/s10654-017-0354-8 |
| 243 | Kaluza J, Håkansson N, Brzozowska A, et al. (2009) Diet quality and mortality: A population-based prospective study of men. Eur J Clin Nutr. 63(4):451-7. http://doi.org/10.1038/sj.ejcn.1602968 |
| 245 | Kant AK, Graubard BI, Schatzkin A (2004) Dietary patterns predict mortality in a national cohort: The National Health Interview Surveys, 1987 and 1992. J Nutr. 134(7):1793-9. http://doi.org/10.1093/jn/134.7.1793 |
| 246 | Kant AK, Schatzkin A, Graubard BI, et al. (2000) A prospective study of diet quality and mortality in women. JAMA. 283(16):2109-15. http://doi.org/10.1001/jama.283.16.2109 |
| 254 | Knoops KTB, de Groot LCPGM, Kromhout D, et al. (2004) Mediterranean diet, lifestyle factors, and 10-year mortality in elderly European men and women: The HALE project. JAMA. 292(12):1433-9. http://doi.org/10.1001/jama.292.12.1433 |

| | |
|---|---|
| 255 | Knoops KTB, de Groot LC, Fidanza F, et al. (2006) Comparison of three different dietary scores in relation to 10-year mortality in elderly European subjects: the HALE project. Eur J Clin Nutr. 60(6):746-55. http://doi.org/10.1038/sj.ejcn.1602378 |
| 262 | Kouris-Blazos A, Gnardellis C, Wahlqvist ML, et al. (1999) Are the advantages of the mediterranean diet transferable to other populations? A cohort study in Melbourne, Australia. Br J Nutr. 82(1):57-61. http://doi.org/10.1017/s0007114599001129 |
| 271 | Lagiou P, Trichopoulos D, Sandin S, et al. (2006) Mediterranean dietary pattern and mortality among young women: A cohort study in Sweden. Br J Nutr. 96(2):384-92. http://doi.org/10.1079/BJN20061824 |
| 280 | Lassale C, Gunter MJ, Romaguera D, et al. (2016) Diet quality scores and prediction of all-cause, cardiovascular and cancer mortality in a pan-european cohort study. PLoS ONE. 11(7). http://doi.org/10.1371/journal.pone.0159025 |
| 284 | Lee MS, Huang YC, Su HH, et al. (2011) A simple food quality index predicts mortality in Elderly Taiwanese. J Nutr Health Aging. 15(10):815-21. http://doi.org/10.1007/s12603-011-0081-x |
| 288 | Lemming EW, Byberg L, Wolk A, et al. (2018) A comparison between two healthy diet scores, the modified Mediterranean diet score and the Healthy Nordic Food Index, in relation to all-cause and cause-specific mortality. Br J Nutr. 119(7):836-46. http://doi.org/10.1017/S0007114518000387 |
| 299 | Liese AD, Krebs-Smith SM, Subar AF, et al. (2015) The Dietary Patterns Methods Project: synthesis of findings across cohorts and relevance to dietary guidance. J Nutr. 145(3):393-402. http://doi.org/10.3945/jn.114.205336 |
| 302 | Limongi F, Noale M, Gesmundo A, et al. (2017) Adherence to the Mediterranean Diet and all-cause mortality risk in an elderly Italian population: Data from the ILSA study. J Nutr Health Aging. 21(5):505-13. http://doi.org/10.1007/s12603-016-0808-9 |
| 304 | Liu L, Nishihara R, Qian Z, et al. (2017) Association between inflammatory diet pattern and risk of colorectal carcinoma subtypes classified by immune responses to tumor. Gastroenterology. 153(6):1517-30.e14. http://doi.org/10.1053/j.gastro.2017.08.045 |
| 312 | Malekshah AFT, Zaroudi M, Etemadi A, et al. (2016) The combined effects of healthy lifestyle behaviors on all-cause mortality: The Golestan cohort study. Arch Iran Med. 19(11):752-61. |
| 320 | Martínez-González MA, Zazpe I, Razquin C, et al. (2015) Empirically-derived food patterns and the risk of total mortality and cardiovascular events in the PREDIMED study. Clin Nutr. 34(5):859-67. http://doi.org/10.1016/j.clnu.2014.09.006 |
| 325 | Masala G, Ceroti M, Pala V, et al. (2007) A dietary pattern rich in olive oil and raw vegetables is associated with lower mortality in Italian elderly subjects. Br J Nutr. 98(2):406-15. http://doi.org/10.1017/S0007114507704981 |
| 330 | McCullough ML, Feskanich D, Rimm EB, et al. (2000) Adherence to the Dietary Guidelines for Americans and risk of major chronic disease in men. Am J Clin Nutr. 72(5):1223-31. http://doi.org/10.1093/ajcn/72.5.1223 |

| | |
|---|---|
| 331 | McCullough ML, Willett WC (2006) Evaluating adherence to recommended diets in adults: the Alternate Healthy Eating Index. Public Health Nutr. 9(1A):152-7. http://doi.org/10.1079/PHN2005938 |
| 332 | McNaughton SA, Bates CJ, Mishra GD (2012) Diet quality is associated with all-cause mortality in adults aged 65 years and older. J Nutr. 142(2):320-5. http://doi.org/10.3945/jn.111.148692 |
| 341 | Menotti A, Puddu PE, Lanti M, et al. (2014) Lifestyle habits and mortality from all and specific causes of death: 40-year follow-up in the italian rural areas of the seven countries study. J Nutr Health Aging. 18(3):314-21. http://doi.org/10.1007/s12603-013-0392-1 |
| 343 | Meyer J, Döring A, Herder C, et al. (2011) Dietary patterns, subclinical inflammation, incident coronary heart disease and mortality in middle-aged men from the MONICA/KORA Augsburg cohort study. Eur J Clin Nutr. 65(7):800-7. http://doi.org/10.1038/ejcn.2011.37 |
| 345 | Michels KB, Wolk A (2002) A prospective study of variety of healthy foods and mortality in women. Int J Epidemiol. 31(4):847-54. http://doi.org/10.1093/ije/31.4.847 |
| 378 | Odegaard AO, Koh WP, Yuan JM, et al. (2014) Dietary patterns and mortality in a Chinese population. Am J Clin Nutr. 100(3):877-83. http://doi.org/10.3945/ajcn.114.086124 |
| 387 | Osler M, Heitmann BL, Gerdes LU, et al. (2001) Dietary patterns and mortality in Danish men and women: A prospective observational study. Br J Nutr. 85(2):219-25. http://doi.org/10.1079/BJN2000240 |
| 388 | Osler M, Heitmann BL, Høidrup S, et al. (2001) Food intake patterns, self rated health and mortality in Danish men and women. A prospective observational study. J Epidemiol Community Health. 55(6):399-403. http://doi.org/10.1136/jech.55.6.399 |
| 389 | Osler M, Schroll M (1997) Diet and mortality in a cohort of elderly people in a North European Community. Int J Epidemiol. 26(1):155-9. http://doi.org/10.1093/ije/26.1.155 |
| 393 | Panizza CE, Shvetsov YB, Harmon BE, et al. (2018) Testing the predictive validity of the healthy eating index-2015 in the multiethnic cohort: Is the score associated with a reduced risk of all-cause and cause-specific mortality? Nutrients. http://doi.org/10.3390/nu10040452 |
| 396 | Park YM, Steck SE, Fung TT, et al. (2016) Mediterranean diet and mortality risk in metabolically healthy obese and metabolically unhealthy obese phenotypes. Int J Obes. 40(10):1541-9. http://doi.org/10.1038/ijo.2016.114 |
| 409 | Prinelli F, Yannakoulia M, Anastasiou CA, et al. (2015) Mediterranean diet and other lifestyle factors in relation to 20-year all-cause mortality: a cohort study in an Italian population. Br J Nutr. 113(6):1003-11. http://doi.org/10.1017/S0007114515000318 |
| 449 | Seymour JD, Calle EE, Flagg EW, et al. (2003) Diet Quality Index as a predictor of short-term mortality in the American Cancer Society Cancer Prevention Study II Nutrition Cohort. Am J Epidemiol. 157(11):980-8. http://doi.org/10.1093/aje/kwg077 |
| 450 | Shah NS, Leonard D, Finley CE, et al. (2018) Dietary Patterns and Long-Term Survival: A Retrospective Study of Healthy Primary Care Patients. Am J Med. 131(1):48-55. http://doi.org/10.1016/j.amjmed.2017.08.010 |

| | |
|---|---|
| 460 | Shivappa N, Hebert JR, Kivimaki M, et al. (2017) Alternative Healthy Eating Index 2010, dietary Inflammatory Index and risk of mortality: results from the Whitehall II cohort study and meta-analysis of previous Dietary Inflammatory Index and mortality studies. Br J Nutr. 118(3):210-21. http://doi.org/10.1017/S0007114517001908 |
| 462 | Shvetsov YB, Harmon BE, Ettienne R, et al. (2016) The influence of energy standardisation on the alternate Mediterranean diet score and its association with mortality in the Multiethnic Cohort. Br J Nutr. 116(9):1592-601. http://doi.org/10.1017/S0007114516003482 |
| 467 | Sjögren P, Becker W, Warensjö E, et al. (2010) Mediterranean and carbohydrate-restricted diets and mortality among elderly men: A cohort study in Sweden. Am J Clin Nutr. 92(4):967-74. http://doi.org/10.3945/ajcn.2010.29345 |
| 475 | Stefler D, Malyutina S, Kubinova R, et al. (2017) Mediterranean diet score and total and cardiovascular mortality in Eastern Europe: the HAPIEE study. Eur J Nutr. 56(1):421-9. http://doi.org/10.1007/s00394-015-1092-x |
| 476 | Stefler D, Pikhart H, Jankovic N, et al. (2014) Healthy diet indicator and mortality in Eastern European populations: Prospective evidence from the HAPIEE cohort. Eur J Clin Nutr. 68(12):1346-52. http://doi.org/10.1038/ejcn.2014.134 |
| 490 | Tognon G, Lissner L, Sæbye D, et al. (2014) The Mediterranean diet in relation to mortality and CVD: A Danish cohort study. Br J Nutr. 111(1):151-9. http://doi.org/10.1017/S0007114513001931 |
| 493 | Tong TYN, Wareham NJ, Khaw KT, et al. (2016) Prospective association of the Mediterranean diet with cardiovascular disease incidence and mortality and its population impact in a non-Mediterranean population: The EPIC-Norfolk study. BMC Med. 14(1). http://doi.org/10.1186/s12916-016-0677-4 |
| 495 | Trichopoulou A (2005) Modified Mediterranean diet and survival: EPIC-elderly prospective cohort study. BMJ. 330(7498):991-5. http://doi.org/10.1136/bmj.38415.644155.8F |
| 497 | Trichopoulou A, Bamia C, Trichopoulos D (2009) Anatomy of health effects of Mediterranean diet: Greek EPIC prospective cohort study. BMJ. 339(7711):26-8. http://doi.org/10.1136/bmj.b2337 |
| 498 | Trichopoulou A, Kouris-Blazos A, Wahlqvist ML, et al. (1995) Diet and overall survival in elderly people. BMJ. 311(7018):1457-60. http://doi.org/10.1136/bmj.311.7018.1457 |
| 504 | Van Dam RM, Li T, Spiegelman D, et al. (2008) Combined impact of lifestyle factors on mortality: Prospective cohort study in US women. BMJ. 337(7672):742-5. http://doi.org/10.1136/bmj.a1440 |
| 512 | Voortman T, Kiefte-de Jong JC, Ikram MA, et al. (2017) Adherence to the 2015 Dutch dietary guidelines and risk of non-communicable diseases and mortality in the Rotterdam Study. Eur J Epidemiol. 32(11):993-1005. http://doi.org/10.1007/s10654-017-0295-2 |
| 515 | Wahlqvist ML, Darmadi-Blackberry I, Kouris-Blazos A, et al. (2005) Does diet matter for survival in long-lived cultures? Asia Pac J Clin Nutr. 14(1):2-6. |

| | |
|---|---|
| 516 | Waijers PMCM, Ocké MC, Rossum CTMv, et al. (2006) Dietary patterns and survival in older Dutch women. Am J Clin Nutr. 83(5):1170-6. http://doi.org/10.1093/ajcn/83.5.1170 |
| 522 | Whalen KA, Judd S, McCullough ML, et al. (2017) Paleolithic and Mediterranean diet pattern scores are inversely associated with all-cause and cause-specific mortality in adults. J Nutr. 147(4):612-20. http://doi.org/10.3945/jn.116.241919 |
| 529 | Yu D, Zhang X, Xiang YB, et al. (2014) Adherence to dietary guidelines and mortality: A report from prospective cohort studies of 134,000 Chinese adults in urban Shanghai. Am J Clin Nutr. 100(2):693-700. http://doi.org/10.3945/ajcn.113.079194 |
| 530 | Yu D, Sonderman J, Buchowski MS, et al. (2015) Healthy Eating and Risks of Total and Cause-Specific Death among Low-Income Populations of African-Americans and Other Adults in the Southeastern United States: A Prospective Cohort Study. PLoS Med. 12(5). http://doi.org/10.1371/journal.pmed.1001830 |
| 535 | Zazpe I, Sánchez-Tainta A, Toledo E, et al. (2014) Dietary patterns and total mortality in a Mediterranean cohort: the SUN Project. J Acad Nutr Diet. 114(1):37-47. http://doi.org/10.1016/j.jand.2013.07.024 |
| 538 | Zhao W, Ukawa S, Okada E, et al. (2018) The associations of dietary patterns with all-cause mortality and other lifestyle factors in the elderly: An age-specific prospective cohort study. Clin Nutr. 38:288-96. http://doi.org/10.1016/j.clnu.2018.01.018 |
| 551 | Abu-Saad K, Endevelt R, Goldsmith R, et al. (2019) Adaptation and predictive utility of a Mediterranean diet screener score. Clin Nutr. http://doi.org/10.1016/j.clnu.2018.12.034 |
| 554 | Chan RSM, Yu BWM, Leung J, et al. (2019) How Dietary Patterns are Related to Inflammaging and Mortality in Community-Dwelling Older Chinese Adults in Hong Kong — A Prospective Analysis. J Nutr Health Aging. 23(2):181-94. http://doi.org/10.1007/s12603-018-1143-0 |
| 557 | Fresán U, Sabaté J, Martínez-Gonzalez MA, et al. (2019) Adherence to the 2015 Dietary Guidelines for Americans and mortality risk in a Mediterranean cohort: The SUN project. Prev Med. 118:317-24. http://doi.org/10.1016/j.ypmed.2018.11.015 |
| 569 | Li Y, Pan A, Wang DD, et al. (2018) Impact of healthy lifestyle factors on life expectancies in the US population. Circulation. 138(4):345-55. http://doi.org/10.1161/CIRCULATIONAHA.117.032047 |
| 570 | Lim J, Lee Y, Shin S, et al. (2018) An association between diet quality index for Koreans (DQI-K) and total mortality in Health Examinees Gem (HEXA-G) study. Nutr Res Pract. 12(3):258-64. http://doi.org/10.4162/nrp.2018.12.3.258 |
| 575 | Neelakantan N, Koh W, Yuan J, et al. (2018) Diet-quality indexes are associated with a lower risk of cardiovascular, respiratory, and all-cause mortality among Chinese adults. J Nutr. 148(8):1323-32. http://doi.org/10.1093/jn/nxy094 |
| 594 | Fresán U, Martínez-González MA, Sabaté J, et al. (2019) Global sustainability (health, environment and monetary costs) of three dietary patterns: Results from a Spanish cohort (the SUN project). BMJ Open. 9(2). http://doi.org/10.1136/bmjopen-2018-021541 |

a. Record identification numbers provided in Datasets C-K.

**Table S4.** Reviews included in the overview of reviews (n=21).

| ID[a] | Citation |
|---|---|
| 900 | Barbaresko J, Koch M, Schulze MB, et al. (2013) Dietary pattern analysis and biomarkers of low-grade inflammation: A systematic literature review. Nutr Rev. 71(8):511-27. http://doi.org/10.1111/nure.12035 |
| 901 | Bhupathiraju SN, Tucker KL (2011) Coronary heart disease prevention: Nutrients, foods, and dietary patterns. Clin Chim Acta. 412(17-18):1493-514. http://doi.org/10.1016/j.cca.2011.04.038 |
| 902 | Dauchet L, Amouyel P, Dallongeville J (2009) Fruits, vegetables and coronary heart disease. Nat Rev Cardiol. 6(9):599-608. http://doi.org/10.1038/nrcardio.2009.131 |
| 903 | Hanson C, Rutten EPA, Wouters EFM, et al. (2013) Diet and vitamin D as risk factors for lung impairment and COPD. Transl Res. 162(4):219-36. http://doi.org/10.1016/j.trsl.2013.04.004 |
| 904 | Hu FB (2002) Dietary pattern analysis: a new direction in nutritional epidemiology. Curr Opin Lipidol. 13(1):3-9. http://doi.org/10.1097/00041433-200202000-00002 |
| 905 | Jacobs DR, Jr., Gross MD, Tapsell LC (2009) Food synergy: an operational concept for understanding nutrition. Am J Clin Nutr. 89(5):1543S-8S. http://doi.org/10.3945/ajcn.2009.26736B |
| 906 | Jacobs Jr DR, Steffen LM (2003) Nutrients, foods, and dietary patterns as exposures in research: A framework for food synergy. Am J Clin Nutr. 78(3 SUPPL.):508S-13S. http://doi.org/10.1093/ajcn/78.3.508S |
| 907 | Kant AK (2004) Dietary patterns and health outcomes. J Am Diet Assoc. 104(4):615-35. http://doi.org/10.1016/j.jada.2004.01.010 |
| 908 | Kastorini CM, Milionis HJ, Esposito K, et al. (2011) The effect of mediterranean diet on metabolic syndrome and its components: A meta-analysis of 50 studies and 534,906 individuals. Journal of the American College of Cardiology. 57(11):1299-313. http://doi.org/10.1016/j.jacc.2010.09.073 |
| 909 | Lee JY, Zhao L, Hwang DH (2010) Modulation of pattern recognition receptor-mediated inflammation and risk of chronic diseases by dietary fatty acids. Nutr Rev. 68(1):38-61. http://doi.org/10.1111/j.1753-4887.2009.00259.x |
| 911 | Mozaffarian D (2016) Dietary and Policy Priorities for Cardiovascular Disease, Diabetes, and Obesity: A Comprehensive Review. Circulation. 133(2):187-225. http://doi.org/10.1161/CIRCULATIONAHA.115.018585 |
| 912 | Mozaffarian D, Appel LJ, Van Horn L (2011) Components of a cardioprotective diet: New insights. Circulation. 123(24):2870-91. http://doi.org/10.1161/CIRCULATIONAHA.110.968735 |
| 913 | Ndanuko RN, Tapsell LC, Charlton KE, et al. (2016) Dietary patterns and blood pressure in adults: A systematic review and meta-analysis of randomized controlled trials. Adv Nutr. 7(1):76-89. http://doi.org/10.3945/an.115.009753 |

| | |
|---|---|
| 914 | Newman AB, Brach JS (2001) Diet and obstructive lung diseases. Epidemiol Rev. 23(2):268-87. http://doi.org/10.1093/oxfordjournals.epirev.a000806 |
| 915 | Nordmann AJ, Suter-Zimmermann K, Bucher HC, et al. (2011) Meta-analysis comparing mediterranean to low-fat diets for modification of cardiovascular risk factors. Am J Med. 124(9):841-51.e2. http://doi.org/10.1016/j.amjmed.2011.04.024 |
| 917 | Ross SA (2010) Evidence for the relationship between diet and cancer. Exp Oncol. 32(3):137-42. |
| 918 | Serra-Majem L, Roman B, Estruch R (2006) Scientific evidence of interventions using the Mediterranean Diet: A systematic review. Nutr Rev. 64(SUPPL. 1):S27-S47. http://doi.org/10.1111/j.1753-4887.2006.tb00232.x |
| 919 | Siervo M, Lara J, Chowdhury S, et al. (2015) Effects of the dietary approach to stop hypertension (DASH) diet on cardiovascular risk factors: A systematic review and meta-analysis. Br J Nutr. 113(1):1-15. http://doi.org/10.1017/S0007114514003341 |
| 920 | Tapsell LC, Neale EP, Satija A, et al. (2016) Foods, nutrients, and dietary patterns: interconnections and implications for dietary guidelines. Adv Nutr. 7(3):445-54. http://doi.org/10.3945/an.115.011718 |
| 921 | Tosti V, Bertozzi B, Fontana L (2018) Health Benefits of the Mediterranean Diet: Metabolic and Molecular Mechanisms. J Gerontol A Biol Sci Med Sci. 73(3):318-26. http://doi.org/10.1093/gerona/glx227 |
| 922 | Willett WC (1994) Diet and health: What should we eat? Science. 264(5158):532-7. http://doi.org/10.1126/science.8160011 |

a. Record identification numbers provided in Datasets A and B.

**Table S5.** Strength of evidence assessed using the Hierarchies of Evidence Applied to Lifestyle Medicine (HEALM) approach[a]

| Q1: Are there established mechanisms of action? | Q2: Are there intervention studies in people that provide evidence of causality/attribution? | Q3: Are there observational studies to establish generalizability to large, populations? | Q4: Are there observational studies to support effects over time periods measured in decades, lifetimes, or generations? | Overall strength of evidence |
|---|---|---|---|---|
| **Dataset A** | **Dataset B** | **Dataset C** | **Dataset C** | **Dataset A+B+C** |
| Quantity<br>Is there evidence from mechanistic studies?<br>**Yes if at least 1 review included at least 3 mechanistic studies.**<br>No if no reviews included at least 3 mechanistic studies.<br><br>*5 reviews included at least 3 mechanistic studies.* | Quantity<br>Is there evidence from intervention studies?<br>**Yes if at least 1 review included at least 3 intervention studies.**<br>No if no reviews included at least 3 intervention studies.<br><br>*21 reviews included at least 3 intervention studies.* | Quantity<br>Is there evidence from large, prospective cohort studies?<br>**Yes if evidence was derived from at least 3 prospective cohort studies that included >1000 participants at follow up.**<br>No if evidence was derived from <3 prospective cohort studies that included >1000 participants at follow up.<br><br>*46 prospective cohort studies with >1000 participants at follow-up.* | Quantity<br>Is there evidence from observational studies conducted over time periods measured in decades, lifetimes, or generations?<br>**Yes if evidence was derived from at least 3 studies with length of follow-up >10 years.**<br>No if evidence was derived from <3 studies with length of follow-up >10 years.<br><br>*31 prospective cohort studies with length of follow-up >10 years.* | |
| Quality | Quality | Quality | Quality | |

| | | | |
|---|---|---|---|
| Was most of the evidence derived from studies with low ROB? Yes if most of the evidence was derived from studies with low ROB in at least 1 review. **No if most of the evidence was derived from studies with low ROB in <1 review.** *Most of the evidence was derived from studies with low ROB in 0 reviews.* | Was most of the evidence derived from studies with low ROB? **Yes if most of the evidence was derived from studies with low ROB in at least 1 review.** No if most of the evidence was derived from studies with low ROB in <1 review. *Most of the evidence was derived from studies with low ROB in 1 review.* | Was most of the evidence derived from studies with low ROB? Yes if >50% of studies had low ROB. **No if <=50% of studies had low ROB.** *0% of studies with low ROB.* | Was most of the evidence derived from studies with low ROB? Yes if >50% of studies had low ROB. **No if <=50% of studies had low ROB.** *0% of studies with low ROB.* |
| Consistency Is there an indication of consistent results between studies? Yes if there was an indication of consistent results between studies for at least 1 outcome in at least 1 review. **No if there was an indication of consistent results between studies in <1 review.** | Consistency Is there an indication of consistent results between studies? **Yes if there was an indication of consistent results between studies for at least 1 outcome in at least 1 review.** No if there was an indication of consistent results between studies in <1 review. | Consistency Is there an indication of consistent results between studies? Yes if I2<50% and/or small differences in effect size and/or overlap in CIs between studies. **No if I2>=50% and/or large differences in effect size and/or NO overlap in CIs between studies.** | Consistency Is there an indication of consistent results between studies? Yes if I2<50% and/or small differences in effect size and/or overlap in CIs between studies. **No if I2>=50% and/or large differences in effect size and/or NO overlap in CIs between studies.** |

| | | | | |
|---|---|---|---|---|
| *There was an indication of consistent results between studies for at least 1 outcome in 0 reviews.* | *There was an indication of consistent results between studies for at least 1 outcome in 4 reviews.* | *'No' based on I2 and large differences in effect size.* | *'No' based on I2 and large differences in effect size.* | |
| Significance<br>Were most of the results significant?<br>Yes if most of the results were significant for at least 1 outcome in at least 1 review.<br>**No if most of the results were significant in <1 review.**<br><br>*Most of the results were significant for at least 1 outcome in 0 reviews.* | Significance<br>Were most of the results significant?<br>**Yes if most of the results were significant for at least 1 outcome in at least 1 review.**<br>No if most of the results were significant in <1 review.<br><br>*Most of the results were significant for at least 1 outcome in 7 reviews.* | Significance<br>Were most of the results significant?<br>**Yes if >50% of results (not studies) were significant (p-value <0.05 and/or CI does NOT cross 1).**<br>No if <=50% of results (not studies) were significant.<br><br>*71% of results were significant.* | Significance<br>Were most of the results significant?<br>**Yes if >50% of results (not studies) were significant (p-value <0.05 and/or CI does NOT cross 1).**<br>No if <=50% of results (not studies) were significant.<br><br>*71% of results were significant.* | |
| Response<br>Yes = 2<br>**Uncertain = 1**<br>No = 0<br><br>*'Yes' for quantity, but 'no' for quality, consistency, and significance.* | Response<br>**Yes = 3**<br>Uncertain = 1<br>No = 0<br><br>*'Yes' for quantity, quality, consistency, and significance.* | Response<br>Yes = 2<br>**Uncertain = 1**<br>No = 0<br><br>*'Yes' for quantity and significance and 'no' for quality and consistency.* | Response<br>Yes = 2<br>**Uncertain = 1**<br>No = 0<br><br>*'Yes' for quantity and significance and 'no' for quality and consistency.* | Overall score<br>6<br><br>Overall rating<br>Grade A: strong/decisive (overall score ≥7)<br>**Grade B: moderate/suggestive (overall score 5 or 6)** |

| | | | | Grade C: insufficient/inconclusive (overall score <5) |
|---|---|---|---|---|
| **Dataset A** | **Dataset B** | **Dataset D** | **Dataset D** | **Dataset A+B+D** |
| Quantity<br>Is there evidence from mechanistic studies?<br>**Yes if at least 1 review included at least 3 mechanistic studies.**<br>No if no reviews included at least 3 mechanistic studies.<br><br>*5 reviews included at least 3 mechanistic studies.* | Quantity<br>Is there evidence from intervention studies?<br>**Yes if at least 1 review included at least 3 intervention studies.**<br>No if no reviews included at least 3 intervention studies.<br><br>*21 reviews included at least 3 intervention studies.* | Quantity<br>Is there evidence from large, prospective cohort studies?<br>**Yes if evidence was derived from at least 3 prospective cohort studies that included >1000 participants at follow up.**<br>No if evidence was derived from <3 prospective cohort studies that included >1000 participants at follow up.<br><br>*25 prospective cohort studies with >1000 participants at follow-up.* | Quantity<br>Is there evidence from observational studies conducted over time periods measured in decades, lifetimes, or generations?<br>**Yes if evidence was derived from at least 3 studies with length of follow-up >10 years.**<br>No if evidence was derived from <3 studies with length of follow-up >10 years.<br><br>*23 prospective cohort studies with length of follow-up >10 years.* | |
| Quality<br>Was most of the evidence derived from studies with low ROB?<br>Yes if most of the evidence was derived from studies | Quality<br>Was most of the evidence derived from studies with low ROB?<br>**Yes if most of the evidence was derived from studies** | Quality<br>Was most of the evidence derived from studies with low ROB?<br>Yes if >50% of studies had low ROB. | Quality<br>Was most of the evidence derived from studies with low ROB?<br>Yes if >50% of studies had low ROB. | |

| | | | |
|---|---|---|---|
| with low ROB in at least 1 review.<br>**No if most of the evidence was derived from studies with low ROB in <1 review.**<br><br>*Most of the evidence was derived from studies with low ROB in 0 reviews.* | **with low ROB in at least 1 review.**<br>No if most of the evidence was derived from studies with low ROB in <1 review.<br><br>*Most of the evidence was derived from studies with low ROB in 1 review.* | **No if <=50% of studies had low ROB.**<br><br>*0% of studies with low ROB.* | **No if <=50% of studies had low ROB.**<br><br>*0% of studies with low ROB.* |
| <u>Consistency</u><br>Is there an indication of consistent results between studies?<br>Yes if there was an indication of consistent results between studies for at least 1 outcome in at least 1 review.<br>**No if there was an indication of consistent results between studies in <1 review.**<br><br>*There was an indication of consistent results between* | <u>Consistency</u><br>Is there an indication of consistent results between studies?<br>**Yes if there was an indication of consistent results between studies for at least 1 outcome in at least 1 review.**<br>No if there was an indication of consistent results between studies in <1 review.<br><br>*There was an indication of consistent results between* | <u>Consistency</u><br>Is there an indication of consistent results between studies?<br>Yes if I2<50% and/or small differences in effect size and/or overlap in CIs between studies.<br>**No if I2>=50% and/or large differences in effect size and/or NO overlap in CIs between studies.**<br><br>*'No' based on I2.* | <u>Consistency</u><br>Is there an indication of consistent results between studies?<br>Yes if I2<50% and/or small differences in effect size and/or overlap in CIs between studies.<br>**No if I2>=50% and/or large differences in effect size and/or NO overlap in CIs between studies.**<br><br>*'No' based on I2.* |

| | | | | |
|---|---|---|---|---|
| *studies for at least 1 outcome in 0 reviews.* | *studies for at least 1 outcome in 4 reviews.* | | | |
| Significance<br>Were most of the results significant?<br>Yes if most of the results were significant for at least 1 outcome in at least 1 review.<br>**No if most of the results were significant in <1 review.**<br><br>*Most of the results were significant for at least 1 outcome in at 0 reviews.* | Significance<br>Were most of the results significant?<br>**Yes if most of the results were significant for at least 1 outcome in at least 1 review.**<br>No if most of the results were significant in <1 review.<br><br>*Most of the results were significant for at least 1 outcome in 7 reviews.* | Significance<br>Were most of the results significant?<br>**Yes if >50% of results (not studies) were significant (p-value <0.05 and/or CI does NOT cross 1).**<br>No if <=50% of results (not studies) were significant.<br><br>*69% of results were significant.* | Significance<br>Were most of the results significant?<br>**Yes if >50% of results (not studies) were significant (p-value <0.05 and/or CI does NOT cross 1).**<br>No if <=50% of results (not studies) were significant.<br><br>*69% of results were significant.* | |
| Response<br>Yes = 2<br>**Uncertain = 1**<br>No = 0<br><br>*'Yes' for quantity, but 'no' for quality, consistency, and significance.* | Response<br>**Yes = 3**<br>Uncertain = 1<br>No = 0<br><br>*'Yes' for quantity, quality, consistency, and significance.* | Response<br>Yes = 2<br>**Uncertain = 1**<br>No = 0<br><br>*'Yes' for quantity and significance and 'no' for quality and consistency.* | Response<br>Yes = 2<br>**Uncertain = 1**<br>No = 0<br><br>*'Yes' for quantity and significance and 'no' for quality and consistency.* | Overall score<br>6<br><br>Overall rating<br>Grade A: strong/decisive (overall score ≥7)<br>**Grade B: moderate/suggestive (overall score 5 or 6)**<br>Grade C: insufficient/inconclusive (overall score <5) |

| Dataset A | Dataset B | Dataset E | Dataset E | Dataset A+B+E |
|---|---|---|---|---|
| Quantity<br>Is there evidence from mechanistic studies?<br>**Yes if at least 1 review included at least 3 mechanistic studies.**<br>No if no reviews included at least 3 mechanistic studies.<br><br>*5 reviews included at least 3 mechanistic studies.* | Quantity<br>Is there evidence from intervention studies?<br>**Yes if at least 1 review included at least 3 intervention studies.**<br>No if no reviews included at least 3 intervention studies.<br><br>*21 reviews included at least 3 intervention studies.* | Quantity<br>Is there evidence from large, prospective cohort studies?<br>**Yes if evidence was derived from at least 3 prospective cohort studies that included >1000 participants at follow up.**<br>No if evidence was derived from <3 prospective cohort studies that included >1000 participants at follow up.<br><br>*13 prospective cohort studies with >1000 participants at follow-up.* | Quantity<br>Is there evidence from observational studies conducted over time periods measured in decades, lifetimes, or generations?<br>**Yes if evidence was derived from at least 3 studies with length of follow-up >10 years.**<br>No if evidence was derived from <3 studies with length of follow-up >10 years.<br><br>*6 prospective cohort studies with length of follow-up >10 years.* | |
| Quality<br>Was most of the evidence derived from studies with low ROB?<br>Yes if most of the evidence was derived from studies with low ROB in at least 1 review. | Quality<br>Was most of the evidence derived from studies with low ROB?<br>**Yes if most of the evidence was derived from studies with low ROB in at least 1 review.** | Quality<br>Was most of the evidence derived from studies with low ROB?<br>Yes if >50% of studies had low ROB.<br>**No if <=50% of studies had low ROB.** | Quality<br>Was most of the evidence derived from studies with low ROB?<br>Yes if >50% of studies had low ROB.<br>**No if <=50% of studies had low ROB.** | |

| | | | |
|---|---|---|---|
| **No if most of the evidence was derived from studies with low ROB in <1 review.**<br><br>*Most of the evidence was derived from studies with low ROB in 0 reviews.* | No if most of the evidence was derived from studies with low ROB in <1 review.<br><br>*Most of the evidence was derived from studies with low ROB in 1 review.* | *0% of studies with low ROB.* | *0% of studies with low ROB.* |
| <u>Consistency</u><br>Is there an indication of consistent results between studies?<br>Yes if there was an indication of consistent results between studies for at least 1 outcome in at least 1 review.<br>**No if there was an indication of consistent results between studies in <1 review.**<br><br>*There was an indication of consistent results between studies for at least 1 outcome in 0 reviews.* | <u>Consistency</u><br>Is there an indication of consistent results between studies?<br>**Yes if there was an indication of consistent results between studies for at least 1 outcome in at least 1 review.**<br>No if there was an indication of consistent results between studies in <1 review.<br><br>*There was an indication of consistent results between studies for at least 1 outcome in 4 reviews.* | <u>Consistency</u><br>Is there an indication of consistent results between studies?<br>Yes if I2<50% and/or small differences in effect size and/or overlap in CIs between studies.<br>**No if I2>=50% and/or large differences in effect size and/or NO overlap in CIs between studies.**<br><br>*'No' based on I2 and large differences in effect size.* | <u>Consistency</u><br>Is there an indication of consistent results between studies?<br>Yes if I2<50% and/or small differences in effect size and/or overlap in CIs between studies.<br>**No if I2>=50% and/or large differences in effect size and/or NO overlap in CIs between studies.**<br><br>*'No' based on I2 and large differences in effect size.* |
| <u>Significance</u> | <u>Significance</u> | <u>Significance</u> | <u>Significance</u> |

| | | | | |
|---|---|---|---|---|
| Were most of the results significant?<br>Yes if most of the results were significant for at least 1 outcome in at least 1 review.<br>**No if most of the results were significant in <1 review.**<br><br>*Most of the results were significant for at least 1 outcome in at 0 reviews.* | Were most of the results significant?<br>**Yes if most of the results were significant for at least 1 outcome in at least 1 review.**<br>No if most of the results were significant in <1 review.<br><br>*Most of the results were significant for at least 1 outcome in 7 reviews.* | Were most of the results significant?<br>**Yes if >50% of results (not studies) were significant (p-value <0.05 and/or CI does NOT cross 1).**<br>No if <=50% of results (not studies) were significant.<br><br>*68% of results were significant.* | Were most of the results significant?<br>**Yes if >50% of results (not studies) were significant (p-value <0.05 and/or CI does NOT cross 1).**<br>No if <=50% of results (not studies) were significant.<br><br>*68% of results were significant.* | |
| <u>Response</u><br>Yes = 2<br>**Uncertain = 1**<br>No = 0<br><br>*'Yes' for quantity, but 'no' for quality, consistency, and significance.* | <u>Response</u><br>**Yes = 3**<br>Uncertain = 1<br>No = 0<br><br>*'Yes' for quantity, quality, consistency, and significance.* | <u>Response</u><br>Yes = 2<br>**Uncertain = 1**<br>No = 0<br><br>*'Yes' for quantity and significance and 'no' for quality and consistency.* | <u>Response</u><br>Yes = 2<br>**Uncertain = 1**<br>No = 0<br><br>*'Yes' for quantity and significance and 'no' for quality and consistency.* | <u>Overall score</u><br>6<br><br><u>Overall rating</u><br>Grade A: strong/decisive (overall score ≥7)<br>**Grade B: moderate/suggestive (overall score 5 or 6)**<br>Grade C: insufficient/inconclusive (overall score <5) |
| **Dataset A** | **Dataset B** | **Dataset F** | **Dataset F** | **Dataset A+B+F** |
| <u>Quantity</u> | <u>Quantity</u> | <u>Quantity</u> | <u>Quantity</u> | |

| | | | |
|---|---|---|---|
| Is there evidence from mechanistic studies? **Yes if at least 1 review included at least 3 mechanistic studies.** No if no reviews included at least 3 mechanistic studies. *5 reviews included at least 3 mechanistic studies.* | Is there evidence from intervention studies? **Yes if at least 1 review included at least 3 intervention studies.** No if no reviews included at least 3 intervention studies. *21 reviews included at least 3 intervention studies.* | Is there evidence from large, prospective cohort studies? Yes if evidence was derived from at least 3 prospective cohort studies that included >1000 participants at follow up. **No if evidence was derived from <3 prospective cohort studies that included >1000 participants at follow up.** *2 prospective cohort studies with >1000 participants at follow-up.* | Is there evidence from observational studies conducted over time periods measured in decades, lifetimes, or generations? Yes if evidence was derived from at least 3 studies with length of follow-up >10 years. **No if evidence was derived from <3 studies with length of follow-up >10 years.** *2 prospective cohort studies with length of follow-up >10 years.* |
| <u>Quality</u> Was most of the evidence derived from studies with low ROB? Yes if most of the evidence was derived from studies with low ROB in at least 1 review. **No if most of the evidence was derived from studies** | <u>Quality</u> Was most of the evidence derived from studies with low ROB? **Yes if most of the evidence was derived from studies with low ROB in at least 1 review.** | <u>Quality</u> Was most of the evidence derived from studies with low ROB? Yes if >50% of studies had low ROB. **No if <=50% of studies had low ROB.** *0% of studies with low ROB.* | <u>Quality</u> Was most of the evidence derived from studies with low ROB? Yes if >50% of studies had low ROB. **No if <=50% of studies had low ROB.** *0% of studies with low ROB.* |

| | | | | |
|---|---|---|---|---|
| **with low ROB in <1 review.**<br><br>*Most of the evidence was derived from studies with low ROB in 0 reviews.* | No if most of the evidence was derived from studies with low ROB in <1 review.<br><br>*Most of the evidence was derived from studies with low ROB in 1 review.* | | | |
| <u>Consistency</u><br>Is there an indication of consistent results between studies?<br>Yes if there was an indication of consistent results between studies for at least 1 outcome in at least 1 review.<br>**No if there was an indication of consistent results between studies in <1 review.**<br><br>*There was an indication of consistent results between studies for at least 1 outcome in 0 reviews.* | <u>Consistency</u><br>Is there an indication of consistent results between studies?<br>**Yes if there was an indication of consistent results between studies for at least 1 outcome in at least 1 review.**<br>No if there was an indication of consistent results between studies in <1 review.<br><br>*There was an indication of consistent results between studies for at least 1 outcome in 4 reviews.* | <u>Consistency</u><br>Is there an indication of consistent results between studies?<br>Yes if I2<50% and/or small differences in effect size and/or overlap in CIs between studies.<br>**No if I2>=50% and/or large differences in effect size and/or NO overlap in CIs between studies.**<br><br>*'No' based on I2 and large differences in effect size.* | <u>Consistency</u><br>Is there an indication of consistent results between studies?<br>Yes if I2<50% and/or small differences in effect size and/or overlap in CIs between studies.<br>**No if I2>=50% and/or large differences in effect size and/or NO overlap in CIs between studies.**<br><br>*'No' based on I2 and large differences in effect size.* | |
| <u>Significance</u><br>Were most of the results significant? | <u>Significance</u><br>Were most of the results significant? | <u>Significance</u><br>Were most of the results significant? | <u>Significance</u><br>Were most of the results significant? | |

| | | | | |
|---|---|---|---|---|
| Yes if most of the results were significant for at least 1 outcome in at least 1 review.<br>**No if most of the results were significant in <1 review.**<br><br>*Most of the results were significant for at least 1 outcome in at 0 reviews.* | **Yes if most of the results were significant for at least 1 outcome in at least 1 review.**<br>No if most of the results were significant in <1 review.<br><br>*Most of the results were significant for at least 1 outcome in 7 reviews.* | **Yes if >50% of results (not studies) were significant (p-value <0.05 and/or CI does NOT cross 1).**<br>No if <=50% of results (not studies) were significant.<br><br>*67% of results were significant.* | **Yes if >50% of results (not studies) were significant (p-value <0.05 and/or CI does NOT cross 1).**<br>No if <=50% of results (not studies) were significant.<br><br>*67% of results were significant.* | |
| Response<br>Yes = 2<br>**Uncertain = 1**<br>No = 0<br><br>*'Yes' for quantity, but 'no' for quality, consistency, and significance.* | Response<br>**Yes = 3**<br>Uncertain = 1<br>No = 0<br><br>*'Yes' for quantity, quality, consistency, and significance.* | Response<br>Yes = 2<br>Uncertain = 1<br>**No = 0**<br><br>*'No' for quantity. Quality, consistency, and significance don't matter if there isn't enough evidence.* | Response<br>Yes = 2<br>Uncertain = 1<br>**No = 0**<br><br>*'No' for quantity. Quality, consistency, and significance don't matter if there isn't enough evidence.* | Overall score<br>4<br><br>Overall rating<br>Grade A: strong/decisive (overall score ≥7)<br>Grade B: moderate/suggestive (overall score 5 or 6)<br>**Grade C: insufficient/inconclusive (overall score <5)** |
| **Dataset A** | **Dataset B** | **Dataset G** | **Dataset G** | **Dataset A+B+G** |
| Quantity<br>Is there evidence from mechanistic studies? | Quantity<br>Is there evidence from intervention studies? | Quantity<br>Is there evidence from large, prospective cohort studies? | Quantity<br>Is there evidence from observational studies conducted over time | |

| | | | |
|---|---|---|---|
| **Yes if at least 1 review included at least 3 mechanistic studies.**<br>No if no reviews included at least 3 mechanistic studies.<br><br>*5 reviews included at least 3 mechanistic studies.* | **Yes if at least 1 review included at least 3 intervention studies.**<br>No if no reviews included at least 3 intervention studies.<br><br>*21 reviews included at least 3 intervention studies.* | Yes if evidence was derived from at least 3 prospective cohort studies that included >1000 participants at follow up.<br>**No if evidence was derived from <3 prospective cohort studies that included >1000 participants at follow up.**<br><br>*1 prospective cohort study with >1000 participants at follow-up.* | periods measured in decades, lifetimes, or generations?<br>Yes if evidence was derived from at least 3 studies with length of follow-up >10 years.<br>**No if evidence was derived from <3 studies with length of follow-up >10 years.**<br><br>*2 prospective cohort studies with length of follow-up >10 years.* |
| Quality<br>Was most of the evidence derived from studies with low ROB?<br>Yes if most of the evidence was derived from studies with low ROB in at least 1 review.<br>**No if most of the evidence was derived from studies with low ROB in <1 review.** | Quality<br>Was most of the evidence derived from studies with low ROB?<br>**Yes if most of the evidence was derived from studies with low ROB in at least 1 review.**<br>No if most of the evidence was derived from studies with low ROB in <1 review. | Quality<br>Was most of the evidence derived from studies with low ROB?<br>Yes if >50% of studies had low ROB.<br>**No if <=50% of studies had low ROB.**<br><br>*0% of studies with low ROB.* | Quality<br>Was most of the evidence derived from studies with low ROB?<br>Yes if >50% of studies had low ROB.<br>**No if <=50% of studies had low ROB.**<br><br>*0% of studies with low ROB.* |

| | | | |
|---|---|---|---|
| *Most of the evidence was derived from studies with low ROB in 0 reviews.* | *Most of the evidence was derived from studies with low ROB in 1 review.* | | |
| Consistency<br>Is there an indication of consistent results between studies?<br>Yes if there was an indication of consistent results between studies for at least 1 outcome in at least 1 review.<br>**No if there was an indication of consistent results between studies in <1 review.**<br><br>*There was an indication of consistent results between studies for at least 1 outcome in 0 reviews.* | Consistency<br>Is there an indication of consistent results between studies?<br>**Yes if there was an indication of consistent results between studies for at least 1 outcome in at least 1 review.**<br>No if there was an indication of consistent results between studies in <1 review.<br><br>*There was an indication of consistent results between studies for at least 1 outcome in 4 reviews.* | Consistency<br>Is there an indication of consistent results between studies?<br>Yes if I2<50% and/or small differences in effect size and/or overlap in CIs between studies.<br>**No if I2>=50% and/or large differences in effect size and/or NO overlap in CIs between studies.**<br><br>*Can't assess consistency when there is only one study.* | Consistency<br>Is there an indication of consistent results between studies?<br>**Yes if I2<50% and/or small differences in effect size and/or overlap in CIs between studies.**<br>No if I2>=50% and/or large differences in effect size and/or NO overlap in CIs between studies.<br><br>*'Yes' based on I2, small differences in effect size, and overlap in CIs.* |
| Significance<br>Were most of the results significant?<br>Yes if most of the results were significant for at least 1 outcome in at least 1 review. | Significance<br>Were most of the results significant?<br>**Yes if most of the results were significant for at least 1 outcome in at least 1 review.** | Significance<br>Were most of the results significant?<br>Yes if >50% of results (not studies) were significant (p-value <0.05 and/or CI does NOT cross 1). | Significance<br>Were most of the results significant?<br>Yes if >50% of results (not studies) were significant (p-value <0.05 and/or CI does NOT cross 1). |

| | | | | |
|---|---|---|---|---|
| **No if most of the results were significant in <1 review.**<br><br>*Most of the results were significant for at least 1 outcome in at 0 reviews.* | No if most of the results were significant in <1 review.<br><br>*Most of the results were significant for at least 1 outcome in 7 reviews.* | **No if <=50% of results (not studies) were significant.**<br><br>*50% of results were significant.* | **No if <=50% of results (not studies) were significant.**<br><br>*50% of results were significant.* | |
| Response<br>Yes = 2<br>**Uncertain = 1**<br>No = 0<br><br>*'Yes' for quantity, but 'no' for quality, consistency, and significance.* | Response<br>**Yes = 3**<br>Uncertain = 1<br>No = 0<br><br>*'Yes' for quantity, quality, consistency, and significance.* | Response<br>Yes = 2<br>Uncertain = 1<br>**No = 0**<br><br>*'No' for quantity. Quality, consistency, and significance don't matter if there isn't enough evidence.* | Response<br>Yes = 2<br>Uncertain = 1<br>**No = 0**<br><br>*'No' for quantity. Quality, consistency, and significance don't matter if there isn't enough evidence.* | Overall score<br>4<br><br>Overall rating<br>Grade A: strong/decisive (overall score ≥7)<br>Grade B: moderate/suggestive (overall score 5 or 6)<br>**Grade C: insufficient/inconclusive (overall score <5)** |
| **Dataset A** | **Dataset B** | **Dataset H** | **Dataset H** | **Dataset A+B+H** |
| Quantity<br>Is there evidence from mechanistic studies?<br>**Yes if at least 1 review included at least 3 mechanistic studies.** | Quantity<br>Is there evidence from intervention studies?<br>**Yes if at least 1 review included at least 3 intervention studies.** | Quantity<br>Is there evidence from large, prospective cohort studies?<br>Yes if evidence was derived from at least 3 prospective cohort studies that | Quantity<br>Is there evidence from observational studies conducted over time periods measured in decades, lifetimes, or generations? | |

| | | | | |
|---|---|---|---|---|
| No if no reviews included at least 3 mechanistic studies.<br><br>*5 reviews included at least 3 mechanistic studies.* | No if no reviews included at least 3 intervention studies.<br><br>*21 reviews included at least 3 intervention studies.* | included >1000 participants at follow up.<br>**No if evidence was derived from <3 prospective cohort studies that included >1000 participants at follow up.**<br><br>*2 prospective cohort studies with >1000 participants at follow-up.* | Yes if evidence was derived from at least 3 studies with length of follow-up >10 years.<br>**No if evidence was derived from <3 studies with length of follow-up >10 years.**<br><br>*2 prospective cohort studies with length of follow-up >10 years.* | |
| <u>Quality</u><br>Was most of the evidence derived from studies with low ROB?<br>Yes if most of the evidence was derived from studies with low ROB in at least 1 review.<br>**No if most of the evidence was derived from studies with low ROB in <1 review.**<br><br>*Most of the evidence was derived from studies with low ROB in 0 reviews.* | <u>Quality</u><br>Was most of the evidence derived from studies with low ROB?<br>**Yes if most of the evidence was derived from studies with low ROB in at least 1 review.**<br>No if most of the evidence was derived from studies with low ROB in <1 review.<br><br>*Most of the evidence was derived from studies with low ROB in 1 review.* | <u>Quality</u><br>Was most of the evidence derived from studies with low ROB?<br>Yes if >50% of studies had low ROB.<br>**No if <=50% of studies had low ROB.**<br><br>*0% of studies with low ROB.* | <u>Quality</u><br>Was most of the evidence derived from studies with low ROB?<br>Yes if >50% of studies had low ROB.<br>**No if <=50% of studies had low ROB.**<br><br>*0% of studies with low ROB.* | |

| | | | |
|---|---|---|---|
| Consistency<br>Is there an indication of consistent results between studies?<br>Yes if there was an indication of consistent results between studies for at least 1 outcome in at least 1 review.<br>**No if there was an indication of consistent results between studies in <1 review.**<br><br>*There was an indication of consistent results between studies for at least 1 outcome in 0 reviews.* | Consistency<br>Is there an indication of consistent results between studies?<br>**Yes if there was an indication of consistent results between studies for at least 1 outcome in at least 1 review.**<br>No if there was an indication of consistent results between studies in <1 review.<br><br>*There was an indication of consistent results between studies for at least 1 outcome in 4 reviews.* | Consistency<br>Is there an indication of consistent results between studies?<br>Yes if I2<50% and/or small differences in effect size and/or overlap in CIs between studies.<br>**No if I2>=50% and/or large differences in effect size and/or NO overlap in CIs between studies.**<br><br>*No MA so no I2. 'No' based on large differences in effect size.* | Consistency<br>Is there an indication of consistent results between studies?<br>Yes if I2<50% and/or small differences in effect size and/or overlap in CIs between studies.<br>**No if I2>=50% and/or large differences in effect size and/or NO overlap in CIs between studies.**<br><br>*No MA so no I2. 'No' based on large differences in effect size.* |
| Significance<br>Were most of the results significant?<br>Yes if most of the results were significant for at least 1 outcome in at least 1 review.<br>**No if most of the results were significant in <1 review.** | Significance<br>Were most of the results significant?<br>**Yes if most of the results were significant for at least 1 outcome in at least 1 review.**<br>No if most of the results were significant in <1 review. | Significance<br>Were most of the results significant?<br>**Yes if >50% of results (not studies) were significant (p-value <0.05 and/or CI does NOT cross 1).**<br>No if <=50% of results (not studies) were significant. | Significance<br>Were most of the results significant?<br>**Yes if >50% of results (not studies) were significant (p-value <0.05 and/or CI does NOT cross 1).**<br>No if <=50% of results (not studies) were significant. |

| | | | | |
|---|---|---|---|---|
| *Most of the results were significant for at least 1 outcome in at 0 reviews.* | *Most of the results were significant for at least 1 outcome in 7 reviews.* | *89% of results were significant.* | *89% of results were significant.* | |
| Response<br>Yes = 2<br>**Uncertain = 1**<br>No = 0<br><br>*'Yes' for quantity, but 'no' for quality, consistency, and significance.* | Response<br>**Yes = 3**<br>Uncertain = 1<br>No = 0<br><br>*'Yes' for quantity, quality, consistency, and significance.* | Response<br>Yes = 2<br>Uncertain = 1<br>**No = 0**<br><br>*'No' for quantity. Quality, consistency, and significance don't matter if there isn't enough evidence.* | Response<br>Yes = 2<br>Uncertain = 1<br>**No = 0**<br><br>*'No' for quantity. Quality, consistency, and significance don't matter if there isn't enough evidence.* | Overall score<br>4<br><br>Overall rating<br>Grade A: strong/decisive (overall score ≥7)<br>Grade B: moderate/suggestive (overall score 5 or 6)<br>**Grade C: insufficient/inconclusive (overall score <5)** |
| **Dataset A** | **Dataset B** | **Dataset I** | **Dataset I** | **Dataset A+B+I** |
| Quantity<br>Is there evidence from mechanistic studies?<br>**Yes if at least 1 review included at least 3 mechanistic studies.**<br>No if no reviews included at least 3 mechanistic studies. | Quantity<br>Is there evidence from intervention studies?<br>**Yes if at least 1 review included at least 3 intervention studies.**<br>No if no reviews included at least 3 intervention studies. | Quantity<br>Is there evidence from large, prospective cohort studies?<br>Yes if evidence was derived from at least 3 prospective cohort studies that included >1000 participants at follow up.<br>**No if evidence was derived from <3 prospective cohort** | Quantity<br>Is there evidence from observational studies conducted over time periods measured in decades, lifetimes, or generations?<br>Yes if evidence was derived from at least 3 studies with length of follow-up >10 years. | |

| | | | |
|---|---|---|---|
| *5 reviews included at least 3 mechanistic studies.* | *21 reviews included at least 3 intervention studies.* | **studies that included >1000 participants at follow up.**<br><br>*1 prospective cohort study with >1000 participants at follow-up.* | **No if evidence was derived from <3 studies with length of follow-up >10 years.**<br><br>*1 prospective cohort study with length of follow-up >10 years.* |
| Quality<br>Was most of the evidence derived from studies with low ROB?<br>Yes if most of the evidence was derived from studies with low ROB in at least 1 review.<br>**No if most of the evidence was derived from studies with low ROB in <1 review.**<br><br>*Most of the evidence was derived from studies with low ROB in 0 reviews.* | Quality<br>Was most of the evidence derived from studies with low ROB?<br>**Yes if most of the evidence was derived from studies with low ROB in at least 1 review.**<br>No if most of the evidence was derived from studies with low ROB in <1 review.<br><br>*Most of the evidence was derived from studies with low ROB in 1 review.* | Quality<br>Was most of the evidence derived from studies with low ROB?<br>Yes if >50% of studies had low ROB.<br>**No if <=50% of studies had low ROB.**<br><br>*0% of studies with low ROB.* | Quality<br>Was most of the evidence derived from studies with low ROB?<br>Yes if >50% of studies had low ROB.<br>**No if <=50% of studies had low ROB.**<br><br>*0% of studies with low ROB.* |
| Consistency<br>Is there an indication of consistent results between studies? | Consistency<br>Is there an indication of consistent results between studies? | Consistency<br>Is there an indication of consistent results between studies? | Consistency<br>Is there an indication of consistent results between studies? |

| | | | |
|---|---|---|---|
| Yes if there was an indication of consistent results between studies for at least 1 outcome in at least 1 review.<br>**No if there was an indication of consistent results between studies in <1 review.**<br><br>*There was an indication of consistent results between studies for at least 1 outcome in 0 reviews.* | **Yes if there was an indication of consistent results between studies for at least 1 outcome in at least 1 review.**<br>No if there was an indication of consistent results between studies in <1 review.<br><br>*There was an indication of consistent results between studies for at least 1 outcome in 4 reviews.* | Yes if I2<50% and/or small differences in effect size and/or overlap in CIs between studies.<br>**No if I2>=50% and/or large differences in effect size and/or NO overlap in CIs between studies.**<br><br>*Can't assess consistency when there is only one study.* | Yes if I2<50% and/or small differences in effect size and/or overlap in CIs between studies.<br>**No if I2>=50% and/or large differences in effect size and/or NO overlap in CIs between studies.**<br><br>*Can't assess consistency when there is only one study.* |
| <u>Significance</u><br>Were most of the results significant?<br>Yes if most of the results were significant for at least 1 outcome in at least 1 review.<br>**No if most of the results were significant in <1 review.**<br><br>*Most of the results were significant for at least 1 outcome in at 0 reviews.* | <u>Significance</u><br>Were most of the results significant?<br>**Yes if most of the results were significant for at least 1 outcome in at least 1 review.**<br>No if most of the results were significant in <1 review.<br><br>*Most of the results were significant for at least 1 outcome in 7 reviews.* | <u>Significance</u><br>Were most of the results significant?<br>**Yes if >50% of results (not studies) were significant (p-value <0.05 and/or CI does NOT cross 1).**<br>No if <=50% of results (not studies) were significant.<br><br>*Only 1 result, and this result was significant.* | <u>Significance</u><br>Were most of the results significant?<br>**Yes if >50% of results (not studies) were significant (p-value <0.05 and/or CI does NOT cross 1).**<br>No if <=50% of results (not studies) were significant.<br><br>*Only 1 result, and this result was significant.* |

| | | | | |
|---|---|---|---|---|
| <u>Response</u><br>Yes = 2<br>**Uncertain = 1**<br>No = 0<br><br>*'Yes' for quantity, but 'no' for quality, consistency, and significance.* | <u>Response</u><br>**Yes = 3**<br>Uncertain = 1<br>No = 0<br><br>*'Yes' for quantity, quality, consistency, and significance.* | <u>Response</u><br>Yes = 2<br>Uncertain = 1<br>**No = 0**<br><br>*'No' for quantity. Quality, consistency, and significance don't matter if there isn't enough evidence.* | <u>Response</u><br>Yes = 2<br>Uncertain = 1<br>**No = 0**<br><br>*'No' for quantity. Quality, consistency, and significance don't matter if there isn't enough evidence.* | <u>Overall score</u><br>4<br><br><u>Overall rating</u><br>Grade A: strong/decisive (overall score ≥7)<br>Grade B: moderate/suggestive (overall score 5 or 6)<br>**Grade C: insufficient/inconclusive (overall score <5)** |
| **Dataset A** | **Dataset B** | **Dataset J** | **Dataset J** | **Dataset A+B+J** |
| <u>Quantity</u><br>Is there evidence from mechanistic studies?<br>**Yes if at least 1 review included at least 3 mechanistic studies.**<br>No if no reviews included at least 3 mechanistic studies.<br><br>*5 reviews included at least 3 mechanistic studies.* | <u>Quantity</u><br>Is there evidence from intervention studies?<br>**Yes if at least 1 review included at least 3 intervention studies.**<br>No if no reviews included at least 3 intervention studies.<br><br>*21 reviews included at least 3 intervention studies.* | <u>Quantity</u><br>Is there evidence from large, prospective cohort studies?<br>**Yes if evidence was derived from at least 3 prospective cohort studies that included >1000 participants at follow up.**<br>No if evidence was derived from <3 prospective cohort studies that included >1000 participants at follow up. | <u>Quantity</u><br>Is there evidence from observational studies conducted over time periods measured in decades, lifetimes, or generations?<br>Yes if evidence was derived from at least 3 studies with length of follow-up >10 years.<br>**No if evidence was derived from <3 studies with length of follow-up >10 years.** | |

| | | | |
|---|---|---|---|
| | | *3 prospective cohort studies with >1000 participants at follow-up.* | *1 prospective cohort study with length of follow-up >10 years.* |
| Quality<br>Was most of the evidence derived from studies with low ROB?<br>Yes if most of the evidence was derived from studies with low ROB in at least 1 review.<br>**No if most of the evidence was derived from studies with low ROB in <1 review.**<br>*Most of the evidence was derived from studies with low ROB in 0 reviews.* | Quality<br>Was most of the evidence derived from studies with low ROB?<br>**Yes if most of the evidence was derived from studies with low ROB in at least 1 review.**<br>No if most of the evidence was derived from studies with low ROB in <1 review.<br>*Most of the evidence was derived from studies with low ROB in 1 review.* | Quality<br>Was most of the evidence derived from studies with low ROB?<br>Yes if >50% of studies had low ROB.<br>**No if <=50% of studies had low ROB.**<br>*0% of studies with low ROB.* | Quality<br>Was most of the evidence derived from studies with low ROB?<br>Yes if >50% of studies had low ROB.<br>**No if <=50% of studies had low ROB.**<br>*0% of studies with low ROB.* |
| Consistency<br>Is there an indication of consistent results between studies?<br>Yes if there was an indication of consistent results between studies for | Consistency<br>Is there an indication of consistent results between studies?<br>**Yes if there was an indication of consistent results between studies for** | Consistency<br>Is there an indication of consistent results between studies?<br>**Yes if I2<50% and/or small differences in effect size and/or overlap in CIs between studies.** | Consistency<br>Is there an indication of consistent results between studies?<br>Yes if I2<50% and/or small differences in effect size and/or overlap in CIs between studies. |

| | | | | |
|---|---|---|---|---|
| at least 1 outcome in at least 1 review.<br>**No if there was an indication of consistent results between studies in <1 review.**<br><br>*There was an indication of consistent results between studies for at least 1 outcome in 0 reviews.* | **at least 1 outcome in at least 1 review.**<br>No if there was an indication of consistent results between studies in <1 review.<br><br>*There was an indication of consistent results between studies for at least 1 outcome in 4 reviews.* | No if I2>=50% and/or large differences in effect size and/or NO overlap in CIs between studies.<br><br>*No MA so no I2. CI only reported for one study. 'Yes' based on small differences in effect size.* | **No if I2>=50% and/or large differences in effect size and/or NO overlap in CIs between studies.**<br><br>*Can't assess consistency when there is only one study.* | |
| <u>Significance</u><br>Were most of the results significant?<br>Yes if most of the results were significant for at least 1 outcome in at least 1 review.<br>**No if most of the results were significant in <1 review.**<br><br>*Most of the results were significant for at least 1 outcome in at 0 reviews.* | <u>Significance</u><br>Were most of the results significant?<br>**Yes if most of the results were significant for at least 1 outcome in at least 1 review.**<br>No if most of the results were significant in <1 review.<br><br>*Most of the results were significant for at least 1 outcome in 7 reviews.* | <u>Significance</u><br>Were most of the results significant?<br>Yes if >50% of results (not studies) were significant (p-value <0.05 and/or CI does NOT cross 1).<br>**No if <=50% of results (not studies) were significant.**<br><br>*0% of results were significant.* | <u>Significance</u><br>Were most of the results significant?<br>Yes if >50% of results (not studies) were significant (p-value <0.05 and/or CI does NOT cross 1).<br>**No if <=50% of results (not studies) were significant.**<br><br>*0% of results were significant.* | |
| <u>Response</u><br>Yes = 2 | <u>Response</u><br>**Yes = 3** | <u>Response</u><br>Yes = 2 | <u>Response</u><br>Yes = 2 | <u>Overall score</u><br>5 |

| | | | | |
|---|---|---|---|---|
| **Uncertain = 1**<br>No = 0<br><br>*'Yes' for quantity, but 'no' for quality, consistency, and significance.* | Uncertain = 1<br>No = 0<br><br>*'Yes' for quantity, quality, consistency, and significance.* | **Uncertain = 1**<br>No = 0<br><br>*'Yes' for quantity and consistency, and 'no' for quality and significance.* | Uncertain = 1<br>**No = 0**<br><br>*'No' for quantity. Quality, consistency, and significance don't matter if there isn't enough evidence.* | Overall rating<br>Grade A: strong/decisive (overall score ≥7)<br>**Grade B: moderate/suggestive (overall score 5 or 6)**<br>Grade C: insufficient/inconclusive (overall score <5) |
| **Dataset A** | **Dataset B** | **Dataset K** | **Dataset K** | **Dataset A+B+K** |
| Quantity<br>Is there evidence from mechanistic studies?<br>**Yes if at least 1 review included at least 3 mechanistic studies.**<br>No if no reviews included at least 3 mechanistic studies.<br><br>*5 reviews included at least 3 mechanistic studies.* | Quantity<br>Is there evidence from intervention studies?<br>**Yes if at least 1 review included at least 3 intervention studies.**<br>No if no reviews included at least 3 intervention studies.<br><br>*21 reviews included at least 3 intervention studies.* | Quantity<br>Is there evidence from large, prospective cohort studies?<br>Yes if evidence was derived from at least 3 prospective cohort studies that included >1000 participants at follow up.<br>**No if evidence was derived from <3 prospective cohort studies that included >1000 participants at follow up.**<br><br>*1 prospective cohort study with >1000 participants at follow-up.* | Quantity<br>Is there evidence from observational studies conducted over time periods measured in decades, lifetimes, or generations?<br>Yes if evidence was derived from at least 3 studies with length of follow-up >10 years.<br>**No if evidence was derived from <3 studies with length of follow-up >10 years.** | |

| | | | |
|---|---|---|---|
| | | | *1 prospective cohort study with length of follow-up >10 years.* |
| Quality<br>Was most of the evidence derived from studies with low ROB?<br>Yes if most of the evidence was derived from studies with low ROB in at least 1 review.<br>**No if most of the evidence was derived from studies with low ROB in <1 review.**<br><br>*Most of the evidence was derived from studies with low ROB in 0 reviews.* | Quality<br>Was most of the evidence derived from studies with low ROB?<br>**Yes if most of the evidence was derived from studies with low ROB in at least 1 review.**<br>No if most of the evidence was derived from studies with low ROB in <1 review.<br><br>*Most of the evidence was derived from studies with low ROB in 1 review.* | Quality<br>Was most of the evidence derived from studies with low ROB?<br>Yes if >50% of studies had low ROB.<br>**No if <=50% of studies had low ROB.**<br><br>*0% of studies with low ROB.* | Quality<br>Was most of the evidence derived from studies with low ROB?<br>Yes if >50% of studies had low ROB.<br>**No if <=50% of studies had low ROB.**<br><br>*0% of studies with low ROB.* |
| Consistency<br>Is there an indication of consistent results between studies?<br>Yes if there was an indication of consistent results between studies for at least 1 outcome in at least 1 review. | Consistency<br>Is there an indication of consistent results between studies?<br>**Yes if there was an indication of consistent results between studies for at least 1 outcome in at least 1 review.** | Consistency<br>Is there an indication of consistent results between studies?<br>Yes if I2<50% and/or small differences in effect size and/or overlap in CIs between studies. | Consistency<br>Is there an indication of consistent results between studies?<br>Yes if I2<50% and/or small differences in effect size and/or overlap in CIs between studies. |

| | | | | |
|---|---|---|---|---|
| **No if there was an indication of consistent results between studies in <1 review.**<br><br>*There was an indication of consistent results between studies for at least 1 outcome in 0 reviews.* | No if there was an indication of consistent results between studies in <1 review.<br><br>*There was an indication of consistent results between studies for at least 1 outcome in 4 reviews.* | **No if I2>=50% and/or large differences in effect size and/or NO overlap in CIs between studies.**<br><br>*Can't assess consistency when there is only one study.* | **No if I2>=50% and/or large differences in effect size and/or NO overlap in CIs between studies.**<br><br>*Can't assess consistency when there is only one study.* | |
| <u>Significance</u><br>Were most of the results significant?<br>Yes if most of the results were significant for at least 1 outcome in at least 1 review.<br>**No if most of the results were significant in <1 review.**<br><br>*Most of the results were significant for at least 1 outcome in at 0 reviews.* | <u>Significance</u><br>Were most of the results significant?<br>**Yes if most of the results were significant for at least 1 outcome in at least 1 review.**<br>No if most of the results were significant in <1 review.<br><br>*Most of the results were significant for at least 1 outcome in 7 reviews.* | <u>Significance</u><br>Were most of the results significant?<br>**Yes if >50% of results (not studies) were significant (p-value <0.05 and/or CI does NOT cross 1).**<br>No if <=50% of results (not studies) were significant.<br><br>*Only 1 result, and this result was significant.* | <u>Significance</u><br>Were most of the results significant?<br>**Yes if >50% of results (not studies) were significant (p-value <0.05 and/or CI does NOT cross 1).**<br>No if <=50% of results (not studies) were significant.<br><br>*Only 1 result, and this result was significant.* | |
| <u>Response</u><br>Yes = 2<br>**Uncertain = 1**<br>No = 0 | <u>Response</u><br>**Yes = 3**<br>Uncertain = 1<br>No = 0 | <u>Response</u><br>Yes = 2<br>Uncertain = 1<br>**No = 0** | <u>Response</u><br>Yes = 2<br>Uncertain = 1<br>**No = 0** | <u>Overall score</u><br>4<br><br><u>Overall rating</u> |

| | | | | |
|---|---|---|---|---|
| *'Yes' for quantity, but 'no' for quality, consistency, and significance.* | *'Yes' for quantity, quality, consistency, and significance.* | *'No' for quantity. Quality, consistency, and significance don't matter if there isn't enough evidence.* | *'No' for quantity. Quality, consistency, and significance don't matter if there isn't enough evidence.* | Grade A: strong/decisive (overall score ≥7)<br>Grade B: moderate/suggestive (overall score 5 or 6)<br>**Grade C: insufficient/inconclusive (overall score <5)** |

CI confidence intervals; MA meta-analysis; NS narrative synthesis; PCA principal component analysis; ROB risk of bias; RRR reduced rank regression.

Dataset A: Overview of 5 reviews that included at least 3 relevant mechanistic studies
Dataset B: Overview of 21 reviews that included at least 3 relevant intervention studies
Dataset C: MA of 49 observational studies (index-based dietary patterns, categorical results)
Dataset D: MA of 33 observational studies (index-based dietary patterns, continuous results)
Dataset E: MA of 13 observational studies (PCA-derived dietary patterns, categorical results)
Dataset F: MA of 3 observational studies (PCA-derived dietary patterns, continuous results)
Dataset G: MA of 2 observational studies (RRR-derived dietary patterns, continuous results)
Dataset H: NS of 2 observational studies (index-based dietary patterns, categorical results)[b]
Dataset I: NS of 1 observational study (index-based dietary patterns, continuous results)[b]
Dataset J: NS of 3 observational studies (PCA-derived dietary patterns, categorical results)[b]
Dataset K: NS of 1 observational study (RRR-derived dietary patterns, continuous results)[b]

a. Selected responses indicated in **bold**. Comments explaining selected responses indicated in *italics*.
b. Studies that were included in the systematic review but could not be included in the MA.
